# Muscle-Derived Cytokines Reduce Growth, Viability and Migratory Activity of Pancreatic Cancer Cells

**DOI:** 10.3390/cancers13153820

**Published:** 2021-07-29

**Authors:** Raphaela Schwappacher, Walburga Dieterich, Dejan Reljic, Christian Pilarsky, Debabrata Mukhopadhyay, David K. Chang, Andrew V. Biankin, Jürgen Siebler, Hans J. Herrmann, Markus F. Neurath, Yurdagül Zopf

**Affiliations:** 1 Medical Department 1, Friedrich-Alexander University Erlangen-Nürnberg, Ulmenweg 18, 91054 Erlangen, Germany; Raphaela.Schwappacher@uk-erlangen.de (R.S.); Walburga.Dieterich@uk-erlangen.de (W.D.); Dejan.Reljic@uk-erlangen.de (D.R.); juergen.siebler@uk-erlangen.de (J.S.); hans.herrmann@uk-erlangen.de (H.J.H.); Markus.Neurath@uk-erlangen.de (M.F.N.); 2 Hector-Center for Nutrition, Exercise and Sports, Medical Department 1, Friedrich-Alexander University Erlangen-Nürnberg, Ulmenweg 18, 91054 Erlangen, Germany; 3 Comprehensive Cancer Center Erlangen-EMN (CCC ER-EMN), Östliche Stadtmauerstraße 30, 91054 Erlangen, Germany; christian.pilarsky@uk-erlangen.de; 4 Department of Surgery, Friedrich-Alexander University Erlangen-Nürnberg, Schwabachanlage 12, 91054 Erlangen, Germany; 5 Biochemistry and Molecular Biology, Mayo Clinic College of Medicine and Science, 4500 San Pablo Road, Jacksonville, FL 32224, USA; mukhopadhyay.debabrata@mayo.edu; 6 Wolfson Wohl Cancer Research Centre, Institute of Cancer Sciences, University of Glasgow, Garscube Estate Switchback Road, Glasgow G61 1QH, UK; David.Chang@glasgow.ac.uk (D.K.C.); Andrew.Biankin@glasgow.ac.uk (A.V.B.); 7 West of Scotland Pancreatic Unit, Glasgow Royal Infirmary, 84 Castle Street, Glasgow G4 0SF, UK

**Keywords:** physical activity, exercise, whole-body electromyostimulation, myokine, pancreatic cancer, proliferation, apoptosis, migration, electric pulse stimulation

## Abstract

**Simple Summary:**

Pancreatic cancer (PC) is a highly fatal malignancy. A major reason for the poor prognosis of patients with PC is the insensitivity to most oncological treatment approaches. It is known that regular exercise reduces the cancer risk. We have already shown that serum from advanced prostate and colon cancer patients after exercise reduces growth and viability of cancer cells. The aim of this study was to identify exercise-induced cytokines in serum from patients with advanced-stage PC that regulate cancer cell proliferation and apoptosis. Our data suggest that a mild resistance exercise training in advanced PC patients induces the release of CXCL1, IL10 and CCL4 from contracting skeletal muscle. We demonstrate that these myokines inhibit growth and migration of PC cells, and induce PC cell death. With this report we provide new knowledge on the cancer-protective function of exercise in PC. Our data strongly support sport therapies for cancer patients.

**Abstract:**

The evidence that regular physical exercise reduces the risk of developing cancer is well described. However, the interaction between physical exercise and cancer is not fully clarified yet. Several myokines released by skeletal muscle appear to have a direct anti-tumour function. There are few data on myokine secretion after exercise in patients with advanced tumours. Pancreatic cancer (PC) is a very aggressive and usually fatal cancer. To investigate the effects of exercise in PC, the blood of advanced-stage PC patients was analysed after 12 weeks of resistance training using whole-body electromyostimulation. After the 12-week training period, the patient serum inhibited the proliferation and the motility of PC cells and enhanced PC cell apoptosis. The impact of exercise training was also investigated in an exercise-mimicking in vitro model using electric pulse stimulation of human myotubes and revealed similar anti-tumour effects on PC cells, clearly indicating direct cancer-protective properties of activated skeletal muscle. Protein and gene expression analyses in plasma from exercise-trained patients and in myotube cultures after in vitro exercise showed that interleukin 10 (IL10), C-X-C motif ligand 1 (CXCL1) and C-C motif chemokine ligand 4 (CCL4) are myokines released from activated skeletal muscle. In accordance with the effects of serum from exercise-trained patients, the supplementation with recombinant IL10, CXCL1 and CCL4 impaired growth and migration of PC cells. Treatment of PC cells with these myokines upregulated caspase 3/7 expression and the cleavage of poly(ADP-ribose) polymerase, leading to enhanced PC cell death. The identification of myokines with anti-tumour properties in advanced-stage PC patients after exercise opens a new perspective in supportive therapy with sports and exercise for cancer patients.

## 1. Introduction

Regular exercise reduces the risk of cancer development, and in cancer survivors lowers the risk of cancer recurrence and mortality [1,2]. The impact of exercise on the body is pleiotropic and our knowledge about how exercise-triggered mechanisms affect cancer risk and progression is incomplete. In recent years it has been shown that skeletal muscle is an endocrine organ with anti-inflammatory and anti-cancer properties [2,3]. Pedersen and colleagues pioneered a new area of research and found that stimulated skeletal muscle secretes humoral factors; hundreds of these so-called myokines are identified so far, although a biological function has been described for only 5% of all known myokines [4,5]. It is reported that specific exercise-related myokines provide health benefits mainly by metabolic improvement in muscle and fat tissue, and by anti-inflammatory action; some of these mechanisms have an indirect impact on cancer [4,6,7]. The prototype myokine is interleukin 6 (IL6) [8,9,10]. IL6 is secreted by the tumour-immune system axis and has pro-inflammatory effects, but also is a myokine that is quickly released by skeletal muscle itself in response to contraction and attenuates inflammation [6]. Over the last 10 years, researchers found significant evidence that myokines can also influence cancer cell growth and viability, e.g., via the induction of apoptosis or the stimulation of the immune system to fight the tumour [2,6,11,12].

A multitude of clinical studies have analysed the effect of physical activity and exercise on cancer risk and cancer progression or recurrence in healthy individuals, early-stage cancer patients and survivors, respectively. Compared to that, only a few mechanistic studies in humans and mice have demonstrated a potential direct role of skeletal muscle and the myokinome on cancer cell growth and viability [2,11,12,13,14,15,16,17,18]. Even less is known about a direct anti-tumour impact of exercise in advanced-stage cancer patients, who often are physically weakened due to a deteriorating muscle status, caused by cancer cachexia and/or the oncological therapy. Are these patients able to effectively exercise in order to secret certain myokines with anti-cancer effects? Cancer patients are in general encouraged to be physically active and regular resistance training is recommended to counteract muscle wasting and loss of muscle strength [19]. Yet, normal resistance exercise with weights may be too exhausting for patients with advanced cancer due to their poor physical status. Whole-body electromyostimulation (WB-EMS) training is an innovative, time-efficient form of resistance exercise; it is less strenuous, and therefore is an advisable exercise alternative for physically limited patients. Recently, we reported that resistance exercise using WB-EMS is well-tolerated and feasible, and yet an effective training method which improves body composition and function by increasing muscle mass in advanced-stage cancer patients under oncological therapy [20].

In a recently published study, we could show that resistance training also has an anti-cancer effect in physically weakened patients with advanced cancer. For the first time we could demonstrate an inhibitory effect of resistance exercise using a WB-EMS training concept on malignant cell growth and viability [21]. Using cancer cell cultures, we found that serum from exercise-trained stage III/IV cancer patients with prostate or colorectal carcinomas decreases human cancer cell viability. Our research further suggested that resistance exercise influences the expression of specific genes that are involved in the regulation of proliferation and apoptosis in human cancer cell lines. A comparative approach using serum from exercise-trained patients and also culture medium from skeletal muscle cells that have been subjected to an exercise-mimicking treatment implied that muscle-derived factors might be responsible for the anti-tumour effects of exercise [21].

Our primary objective in the present report was to identify myokines with anti-cancer properties in the serum of exercising patients with advanced-stage pancreatic cancer (PC). With our data we provide further insight into the relationship between exercise and a highly lethal malignancy.

## 2. Materials and Methods

To investigate myokines with anti-tumour effect and their secretion from skeletal muscle after exercise, plasma and serum of stage III/IV PC patients were analysed. Patients were assigned to either the intervention group with resistance training using WB-EMS or to a non-exercising control group [20].

### 2.1. Ethical Approval

The original pilot study was conducted according to the guidelines of the Declaration of Helsinki, and approved by the Ethics Committee of the Friedrich-Alexander University Erlangen-Nürnberg (Reg.Nr.155_13B, 16 July 2013). It is registered at clinicaltrials.gov (NCT02293239).

### 2.2. Clinical Study Design, Patient Recruitment and Exercise Intervention 

**Figure 1:**
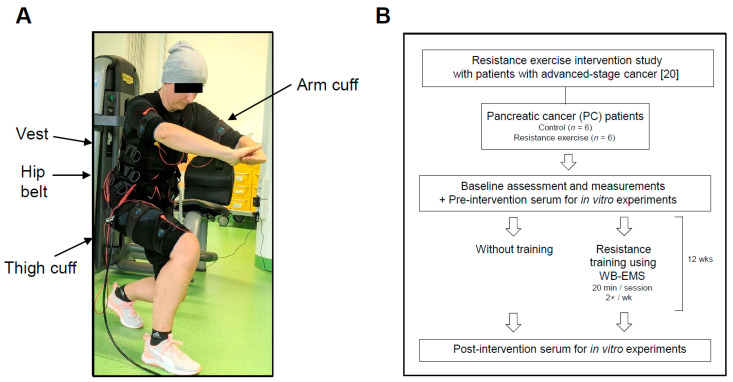
Resistance training using whole-body electromyostimulation (WB-EMS) and study flowchart. (**A**) The picture displays a patient during a 20 min WB-EMS training session. The exercising person wore a vest, a hip belt, and upper-arm and -thigh cuffs with integrated electrodes (for more details, please refer to [21]). Bipolar impulses simultaneously addressed eight large muscle groups. (**B**) Study overview. The PC patients participated in our previous pilot study “Effects of whole-body electromyostimulation combined with individualised nutritional support on body composition in patients with advanced cancer: a controlled pilot trial” [20].

The study protocol and patient recruitment of the original study are described in detail in Schink et al. [20]. In brief, patients (18 years and older), who were diagnosed with a stage III/IV solid tumour disease undergoing oncological therapy, and a performance index (Karnofsky) between 100% and 60%, were included. The baseline characteristics of the patients were assessed, and they were allocated to an exercise group using a WB-EMS training concept for 12 weeks or to a non-exercising control group. Throughout the study, all participants were counselled by a dietitian to achieve a daily protein supply of >1.0 g/kg bodyweight according to the nutritional international guideline; patients with decreased food intake, due to the disease or the oncological therapy, received medical nutritional therapy. The exercise group attended WB-EMS training twice a week (20 min/training) for 12 weeks. Via electrodes, WB-EMS administers low-frequency current impulses (<100 Hz) with a low current intensity (<100 mA) [22]. The patients wore a vest, upper-arm and -thigh cuffs, and a hip belt with integrated electrodes (miha bodytec GmbH, Gersthofen, Germany). The muscles of the upper arms, upper back, latissimus dorsi, chest, abdomen, lower back, buttocks and thighs were stimulated by WB-EMS (Figure 1A) [21]. The electric stimulation induced a periodic stimulation (bipolar impulses: frequency 85 Hz, pulse width 350 μs) with a 6 s impulse phase followed by 4 s rest. The current intensity was set to trigger a noticeable muscle contraction, up to a threshold before inducing discomfort/pain. The current intensity was individually adapted for each muscle region. The individual and local adaptations are highly relevant because of current tolerance limits and differences in subcutaneous fat thickness of each patient [22]. The PC patients in the intervention group performed 22.3 ± 4.3 WB-EMS training sessions out of the scheduled 24 sessions; in one experiment we used serum of exercising and non-exercising patients with advanced gastric cancer, prostate cancer or colorectal cancer (exercising gastric cancer patients attended 21.5 ± 2.2 sessions, exercising prostate cancer patients 20.8 ± 2.6 sessions, and exercising colorectal cancer patients 20.5 ± 2.4 sessions (please see Supplemental Table S1 and [21]). The WB-EMS training was individually supervised by experienced physiotherapists and included light physical exercises [20].

### 2.3. Patient Serum

Blood samples were collected at the beginning of the trial (pre), after 6 weeks of intervention (mid) and at trial end after 12 weeks of intervention (post). The time between the administration of chemotherapy and the blood collection was at least 3 days, except for one exercising patient in whom chemotherapy was administered the day before the blood collection at study entry. In the exercise group the blood withdrawal was done 60 min after the training session using WB-EMS (mid and post). Blood samples were centrifuged and serum and plasma were stored at −80 °C. For the in vitro study, we used serum from stage III/IV pancreatic cancer patients (exercise group *n* = 6, control group *n* = 6; Table 1) under anti-cancer therapy. One experiment was done with serum from patients with stage IV gastric cancer (exercise group *n* = 6, control group *n* = 6; Supplemental Table S1), stage III/IV prostate cancer (exercise group *n* = 8, control group *n* = 10 [21]) or stage III/IV colorectal cancer (exercise group *n* = 6, control group *n* = 6 [21]).

### 2.4. Cell Culture

All cells were cultivated in monolayers at 37 °C in 5% CO_2_ in a humidified incubator. The human pancreatic ductal adenocarcinoma cell line Panc1 (Sigma-Aldrich, Munich, Germany) was cultured in DMEM (Gibco, Thermo-Fisher Scientific, Waltham, MA, USA), 10% FCS Superior (Merck KGaA, Darmstadt, Germany) and 100 IU/mL penicillin/100 µg/mL streptomycin (Gibco). The cells (RRID: CVCL_0480) were kept in culture for no longer than 30 passages. The human pancreatic ductal adenocarcinoma cell line HPAC (ATCC #2119 (LGC Standards GmbH, Wesel, Germany)) was maintained in Ham F12 (Gibco), 10% FCS Superior and penicillin/streptomycin (RRID: CVCL_3517; cells were kept in culture for no longer than 30 passages). The murine pancreatic cancer cell line TB32047 was cultured in DMEM, 10% FCS Superior and penicillin/streptomycin (RRID: CVCL_3517; kept in culture for up to 30 passages). The human pancreatic ductal adenocarcinoma cells PaCaDD119 were maintained in medium consisting of DMEM + 20% FCS (Invitrogen #A3160501, Thermo-Fisher) and keratinocyte serum-free medium and supplements (Thermo-Fisher, mixing ration 2:1, plus penicillin/streptomycin (RRID: CVCL_1848; kept in culture for up to 20 passages) [23]. The human pancreatic ductal adenocarcinoma PC cells Mayo4636 were cultured in Hepes-buffered DMEM/Ham F12, 10% FCS (Hyclone^TM^ #SH30084.03, GE Healthcare, Chicago, IL, USA) and penicillin/streptomycin (cells were kept in culture for no longer than 20 passages) [24]. The human pancreatic ductal adenocarcinoma PC cells TKCC10 (squamous subtype) were maintained as described in Hardie et al. and kept in culture for up to 20 passages [25,26]. The human pancreatic duct epithelial cells HPDE were cultured in keratinocyte serum-free medium and supplements (RRID: CVCL_4376; kept in culture for up to 20 passages). The human embryonic kidney cell line HEK293T was provided by Dr. A. Kremer (Medical Department 1, Friedrich-Alexander University Erlangen-Nürnberg, Erlangen, Germany) and were cultured in DMEM, 10% FCS Superior and penicillin/streptomycin (RRID: CVCL_0045; cells were kept in culture for no longer than 30 passages). The murine embryonic fibroblasts 3T3L1 (Sigma-Aldrich) were maintained in DMEM, 10% FCS Superior and penicillin/streptomycin (RRID: CVCL_0123; kept in culture for up to 10 passages). Primary human skeletal muscle myoblasts (HSMMs) were purchased from Lonza (Basel, Switzerland) and cultured according to manufacturer’s protocol. The murine myoblast cell line C2C12 (DSMZ, Braunschweig, Germany) was cultured in DMEM, 10% FCS Superior and penicillin/streptomycin (RRID: CVCL_0188; kept in culture for up to 15 passages). All cell cultures were mycoplasma-free.

### 2.5. Human Cancer Cell Growth and Apoptosis Assays

3–5 × 10^3^/well or 2.5–3 × 10^4^/well PC cells (Panc1, HPAC, PaCaDD119, Mayo4636, TKCC10), non-malignant pancreatic cells (HPDE) or non-tumour control cells (HEK293T, 3T3L1) were plated into 96-well or 48-well plates, respectively, and after overnight incubation, cells (except of HPDE) were serum-deprived (0.1% FCS) for 18–24 h. Then, cells were stimulated with medium containing 10% patient serum, with EPS-conditioned medium from differentiated myotubes (DMEM/2% FCS plus 10% human serum from healthy individuals) or with recombinant proteins for 24–96 h. Determination of cell growth, apoptosis and viability (BrdU incorporation, total cell number, number of apoptotic cells, DNA fragmentation) was done as described earlier [21]. Of note, the growth response of the tested PC and non-malignant cells did not differ when cultured with human pre-intervention serum compared to bovine serum.

### 2.6. Colony Formation Assay

An amount of 1.2–2.5 × 10^3^/well PC cells (Panc1, PaCaDD119) were seeded into 12-well plates and after overnight incubation, cells were stimulated with medium containing 2–5% FCS with or without recombinant proteins for 7–10 d. Medium was changed every other day. After fixation and staining of the cells with 0.2% crystal violet, colony formation was determined by counting of colonies (n cells > 50). Pictures were taken with the EZ4W stereo microscope (Leica, Wetzlar, Germany) with identical settings (20× magnification, illumination intensity of 3). Images were processed using Photoshop CS5 (Adobe Inc., San Jose, CA, USA; sizing, contrast and brightness adjustment with identical settings for image acquisition of all samples in a given experiment). Additionally, optical density (590 nm) was measured after extraction of the stain using 10% acetic acid.

### 2.7. Scratch Wounding Assay

1.2 × 10^3^ Panc1 cells per well were seeded into 48-well plates and cultivated until they reached at least 95% confluency. Then, cells were serum-deprived for 18 h. Scratch wounding was done using a 100 µL pipette tip and cells were stimulated either with medium containing 10% serum from WB-EMS or control patients, or with medium containing 5% FCS with or without recombinant proteins. Pictures of the wound were taken directly after scratching (0 h) and after 24 h using the EVOS digital inverted microscope (AMG, Mill Creek, WA, USA) with a 4-fold magnification and 40% brightness. Images were processed using Photoshop CS5 (sizing, contrast and brightness adjustment with identical settings for all samples in a given experiment). The width of the scratch wound (six measurements along the scratch per picture) was quantified using ImageJ software (National Institute of Health, Bethesda, MD, USA; SCR_003070).

### 2.8. Electric Pulse Stimulation

1.5 × 10^5^/well primary human skeletal muscle myoblasts (HSMMs) or 2.5 × 10^5^/well murine myoblasts (C2C12) were seeded in 6-well plates. After reaching 70–90% confluency, HSMMs or C2C12 myoblasts, were incubated for 3–4 d with DMEM/Ham F12 (Gibco) or DMEM, respectively, both supplemented with 2% Hyclone^TM^ horse serum (GE Healthcare, Chicago, IL, USA) for in vitro differentiation into myotubes. Effective myogenic differentiation was verified by the grade of myoblast fusion and myogenin expression. Electric pulse stimulation was performed with differentiated myotubes as described in Schwappacher et al. [21]. EPS efficiency was examined via quantitative RT-PCR (mRNA expression of CXCL5, PPARGC1A and MSTN). 

### 2.9. Quantitative Reverse Transcription PCR (qRT-PCR)

Total RNA was isolated from cells using QIAzol^®^ reagent (Qiagen, Venlo, The Netherlands) according to manufacturer’s instructions. 0.5–1 µg was transcribed into cDNA using the iScript^TM^ synthesis kit (Bio-Rad, Hercules, CA, USA). Quantitative RT-PCR was performed using the CFX Connect Real-Time System and CFX Manager software (Bio-Rad). The reference genes hypoxanthine phosphoribosyltransferase 1 (HPRT1), glyceraldehyde-3-phosphate dehydrogenase (GAPDH) and/or ribosomal protein lateral stalk subunit P0 (RPLP0) were used to calculate relative mRNA levels with the 2^−∆∆CT^ method.

### 2.10. Primer Sequences

Oligonucleotide synthesis was done by Metabion International AG (Planegg, Germany). For information about primer sequences for the analysis of EPS efficiency, and the expression of myokines and exercise/myokine-responsive genes please refer to Schwappacher et al. [21]. Additionally, the following primers were used: human IL10 (QT00041685, Qiagen); human CXCL1 (f) 5′-GTCCGTGGCCACTGAACTG-3′ and (r) 5′-GGGGATGCAGGATTGAGGC-3′; human CCL4 (QT01008070, Qiagen); human FNDC5 (f) 5′-TGCACTCAGAGGGATGACTG-3′ and (r) 5′-CTTCTTGCAAGGCTGGAAAC-3′; human SPARC (f) 5′-GAGATAGACCCAGCCCAGAG-3′ and (r) 5′-AGAAGATCCAGGCCCTCATG-3′.

### 2.11. Cytokine Array

For simultaneous detection of cytokines/myokines in the pre- and post-intervention plasma pools of exercising (*n* = 6) and non-exercising control patients (*n* = 6) with advanced PC, a Human Cytokine Antibody Array (80 targets; Abcam, Cambridge, UK) was carried out. The assay was conducted according to manufacturer’s instructions. The Amersham 600 Imager (General Electric, Pittsburgh, PA, USA) was used for chemiluminescence detection. Signals were analysed with ImageJ software. Normalisation of the raw densitometry data was done according to manufacturer’s recommendations. The mean density of the negative controls (background) was subtracted from the density of each cytokine spot; afterwards, the values were normalised to the mean positive control signal (for each membrane). The values of the pre-intervention plasma pools from both patient groups were compared and the cytokines with a difference of <20% in these values were selected. Then, the ratios between the values of pre- and post-intervention plasma pools within each patient cohort were determined. Additionally, the ratios between the values of the post-intervention plasma pools of both patient groups were calculated. Differences of >20% were considered significant and the respective cytokines were subjected to further analysis. For details regarding the cytokine array with medium from untreated or EPS-treated primary human myotubes please refer to Schwappacher et al. [21]. For the normalised densitometry data please refer to the Supplemental Tables S2 and S3.

### 2.12. Cytokine Enzyme-Linked Immunosorbent Assay (ELISA)

For measurement of human CXCL1/Groα and human CCL4/Mip1β in the plasma of non-exercising and exercising PC patients, DuoSet ELISA (R & D Systems, Minneapolis, MN, USA) was used according to manufacturer’s instructions. Measurement of human IL10 was performed with Ready-SET-Go! ELISA (Invitrogen, Carlsbad, CA, USA) according to manufacturer’s protocol. For each cytokine the ELISA analysis was done twice.

### 2.13. Immunoblotting

0.6–1 × 10^5^/well PC cells (Panc1, PaCaDD119) were seeded into 12-well plates and after overnight incubation, cells were serum-starved for 18–24 h. Cells were treated with medium containing 20 ng/mL CXCL1, IL10 and CCL4 or BSA as control for 48–72 h. Cells were lysed in cell lysis buffer (0.5% IGEPAL^®^ CA-630, 150 mM NaCl, 20 mM Tris pH 7.4, protease inhibitors (Protease Inhibitor Cocktail Set I, Calbiochem, Merck KGaA), 10 mM phenylmethylsulfonyl fluoride and phosphatase inhibitors (PhosSTOP, Roche Diagnostics, Penzberg, Germany). Separation of the cell extracts was done by sodium dodecyl sulfate (SDS)-polyacrylamide gel electrophoresis. Immunoblotting was carried out with the polyclonal rabbit antibody specific for poly(ADP-ribose) polymerase (PARP; full length, 116 kDa and cleaved PARP large fragment, 89 kDa; Cell Signaling Technologies, Danvers, MA, USA; dilution 1:1000; RRID:AB_2160739). Additionally, the cell extracts were examined by immunoblotting using the monoclonal rabbit antibodies specific for caspase 3 and 7 (both from Cell Signaling Technologies; dilution 1:2000; RRID: AB_2798429, RRID: AB_2687912) and the monoclonal mouse antibody against α-actin (Santa Cruz Biotechnology, Dallas, TX, USA; dilution 1:5000; RRID: AB_626632). The membranes (Immobilon-P Membrane, PVDF, 0.45 µm, Merck KGaA) were analysed with the Amersham 600 Imager using horseradish peroxidase-conjugated secondary antibodies (Jackson ImmunoResearch Laboratories, West Grove, PA, USA) and enhanced chemiluminescence (Clarity Western ECL Substrate, Bio-Rad). The signals were quantified with ImageJ software. 

### 2.14. Statistical Analysis

**Figure 2:**
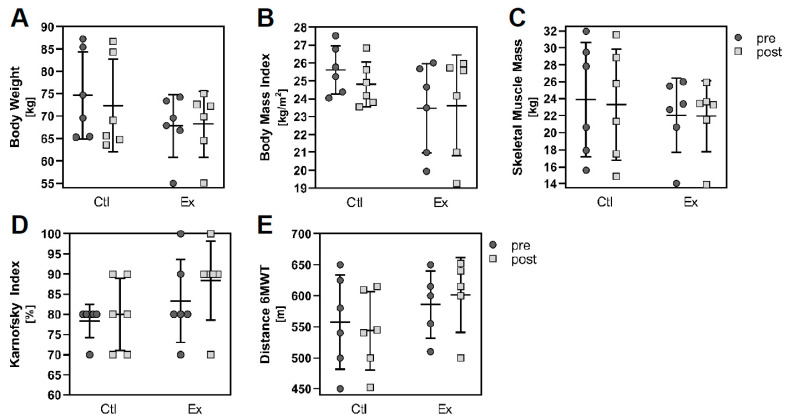
Pre- and post-intervention data regarding the performance status, physical function and body parameters of the two study groups. Graphs display mean (SD), control group *n* = 6, exercise group *n* = 6 (except for 6min-walking distance: control group *n* = 5, exercise group *n* = 5). Statistical analysis was done using paired two-tailed *t*-test and Student’s *t*-test, Wilcoxon signed-rank and Mann–Whitney test.

Study group differences at baseline in Table 1 and Supplemental Table S1 were examined by independent samples *t*-test or Mann–Whitney test. Statistical analysis for body weight, body mass index and skeletal muscle mass in Figure 2 was performed by paired *t*-test (post vs. pre, within the exercise group) and Student’s *t*-test (post, between the groups); statistical analysis for Karnofsky index and 6 min-walking test was done using Wilcoxon signed-rank test (post vs. pre, within the exercise group) and Mann–Whitney test (post, between the groups). For statistical analysis of BrdU uptake, DNA fragmentation and gene expression after stimulation with patient serum, two-tailed paired *t*-test (post vs. pre, within a patient group) or Student’s *t*-test (post, between the groups) were applied. For evaluation of cell counting, Trypan blue exclusion experiments and scratch-wounding assays, Wilcoxon signed-rank test (post vs. pre, within a patient group) and Mann–Whitney test (post, between the groups) were used. Student’s *t*-test was applied for data analysis of BrdU incorporation, DNA fragmentation, gene expression, protein expression (immunoblot densitometry) and OD measurement (colony formation) after stimulation with EPS-conditioned myotube medium or with recombinant proteins. Cell counting, numbers of colonies (colony formation) experiments and scratch-wounding assays after treatment with EPS medium or recombinant proteins were evaluated using Mann–Whitney test. Graphs depict the mean (SD). The respective number of experiments is stated in the Figure legends. Statistical analysis was performed using GraphPad Prism 8.1.2 (GraphPad Software, San Diego, CA, USA; RRID: SCR_002798). *p* values of <0.05 were considered statistically significant.

## 3. Results

### 3.1. Study Design and Patient Data

In order to perform a deeper assessment of the anti-cancer effects of exercise on tumour cells in advanced cancer disease, we used plasma/serum from patients with stage III/IV PC [20]. Patients with advanced PC are very prone to develop cancer cachexia; cachexia-induced muscle wasting strongly limits the physical performance of the affected patients [27]. The study patients participated in a 12-week exercise training using WB-EMS (2×/week, 20 min/session), a form of resistance exercise which can be performed even by very weak patients. The exercise intervention was combined with a high-protein diet according to the international guidelines of nutrition therapy. Figure 1 displays a patient during WB-EMS training and the study flowchart (six PC patients with exercise, six non-exercising PC patients as controls). Table 1 specifies the patients’ baseline demographic and disease characteristics. There were no significant differences between the two study groups regarding body parameters, disease-related weight loss, performance status (Karnofsky index) and physical functioning (6 min-walking distance). Blood parameters did not significantly differ; of note, two of the control patients had a substantially higher blood level of C-reactive protein at trial entry.

The physical status as well as several body parameters improved or at least stabilised in the resistance exercising group after the intervention period of 12 weeks (Figure 2). Performance and physical function increased in the exercising patients (Karnofsky index: pre 83.3% (mean) ± 10.3% (SD), post 88.33 ± 9.83%, Figure 2D; 6 min-walking distance: pre 586.0 ± 54.48 m, post 601.4 ± 60.25 m, Figure 2E), in contrast to the non-exercising controls (Karnofsky index: pre 78.3 ± 4.08%, post 80.0 ± 8.94%; 6-min-walking distance: pre 557.5 ± 75.88 m, post 543.7 ± 62.95 m). Body weight (pre 67.83 ± 6.92 kg, post 68.23 ± 7.35 kg, Figure 2A), body mass index (pre 23.46 ± 2.50 kg/m^2^, post 23.62 ± 2.82 kg/m^2^, Figure 2B) and skeletal muscle mass (pre 22.04 ± 4.38 kg, post 21.95 ± 4.18 kg, Figure 2C) remained stable in the exercising patients during the 12 week intervention; however, these parameters deteriorated in the non-exercising control group (body weight: pre 74.63 ± 9.73 kg, post 72.37 ± 10.38; body mass index: pre 25.62 ± 1.36 kg/m^2^, post 24.81 ± 1.25 kg/m^2^; skeletal muscle mass: pre 23.90 ± 6.73 kg, post 23.32 ± 6.53 kg). Yet, the number of patients per group was too small in order to reach statistical significance.

### 3.2. Exercise Stimuli Affect PC Cell Growth and Viability 

**Figure 3:**
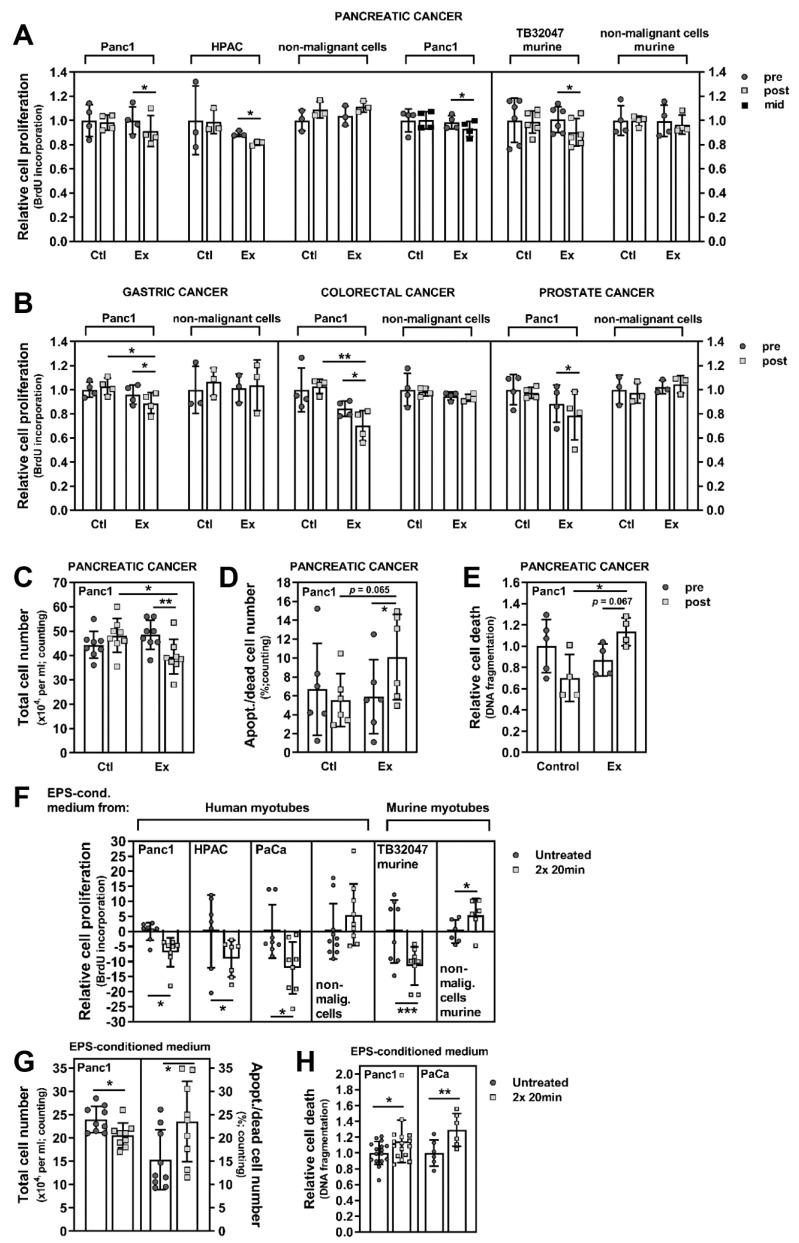
In vivo and in vitro exercise stimuli influence growth and death of PC cells. (**A**) The PC cell lines Panc1, HPAC1 and TB32047, and non-malignant cells (HEK293T, 3T3L1) were serum-starved in medium containing 0.1% FCS for 18–24 h. Then, the cells were stimulated for 96 h with 10% serum from stage III/IV PC patients taken at study entry (pre), after a 6-week (mid) or after a 12-week (post) WB-EMS resistance exercise regimen (control group, Ctl; exercising group, Ex). Cell proliferation was measured and is shown relative to the values after stimulation with a pre-intervention serum pool from control patients (*n* = 6). The graph depicts the mean (SD); number of independent experiments and passage number (p#) at which the experiments were performed: Panc1 *n* = 4 (p# 2–17), HPAC *n* = 3 (p# 3–14), 293T *n* = 3 (p# 21–29), Panc1 (mid) *n* = 4 (p# 2–18), TB32047 *n* = 6 (p# 5–14), 3T3L1 *n* = 4 (p# 2–10). *p* values for non-malignant control cells upon exercise serum post vs. pre: 293T *p* = 0.0656 (Ex pre 1.038 ± 0.08, Ex post 1.112 ± 0.046, Ctl pre 1.00 ± 0.086, Ctl post 1.088 ± 0.069), 3T3L1 *p* = 0.7018 (Ex pre 0.994 ± 0.13, Ex post 0.965 ± 0.078, Ctl pre 1.00 ± 0.123, Ctl post 0.996 ± 0.041). (**B**) Panc1 and non-malignant cells were serum-starved and incubated for 96 h with pre- and post-intervention serum pools from exercising or control patients with advanced gastric (Ex *n* = 6, Ctl *n* = 6), colorectal (Ex *n* = 6, Ctl *n* = 6) or prostate tumours (Ex *n* = 8, Ctl *n* = 10). Then, BrdU incorporation was measured. Additional statistical analysis of the effects of post-intervention sera of exercising vs. control patients on Panc1 cells: gastric *p* = 0.0486; colorectal *p* = 0.0034; prostate *p* = 0.0498. Statistical analysis of pre- vs. post-exercise serum incubation on 293T cells: gastric *p* = 0.6824 (Ex pre 1.011 ± 0.118, Ex post 1.037 ± 0.209, Ctl pre 1.00 ± 0.196, Ctl post 1.064 ± 0.117); colorectal *p* = 0.5426 (Ex pre 0.947 ± 0.033, Ex post 0.933 ± 0.028, Ctl pre 1.00 ± 0.136, Ctl post 0.983 ± 0.035); prostate *p* = 0.6026 (Ex pre 1.022 ± 0.054, Ex post 1.044 ± 0.072, Ctl pre 1.00 ± 0.119, Ctl post 0.973 ± 0.086). Mean (SD); number of independent experiments and passage number: gastric Panc1 *n* = 4 (p# 2–15), 293T *n* = 3 (p# 10–21); colorectal Panc1 *n* = 4 (p# 20–28), 293T *n* = 4 (p# 11–20); prostate Panc1 *n* = 4 (p# 3–17), 293T *n* = 3 (p# 12–21). (**C**,**D**) Starved Panc1 were treated with serum pools from exercising or control PC patients. After 96 h, cell viability was examined by determination of total cell numbers ((**C**); numbers per mL × 10^4^; 8 independent experiments, p# 3–12) and the number of apoptotic/dead cells ((**D**); % of total cell number; additional statistical analysis between the groups after post-intervention serum incubation, *p* = 0.0649; mean (SD), 6 independent experiments, p# 3–12). (**E**) Apoptotic DNA fragmentation in Panc1 cells was determined after 48 h of treatment with exercise serum (shown relative to pre-intervention control serum values; additional statistical analysis between the groups after post-intervention serum incubation, *p* = 0.0147). Mean (SD) of 4 independent experiments, p# 3–16. (**F**) PC cells (Panc1, HPAC, PaCaDD119, TB32047) and non-malignant cells (HEK293T, 3T3L1) were serum-starved and treated with EPS-conditioned medium from in vitro-differentiated human or murine myotubes for 48 h, and cell proliferation was measured. The graph displays the relative proliferation of untreated cells as 0%, and the percentage difference in proliferation after treatment with EPS-conditioned compared to unconditioned medium. *p* values for non-cancer control cells (293T) treated vs. untreated: *p* = 0.6044; 3T3L1, *p* = 0.0377. Mean (SD), number of independent experiments and passage number: Panc1 *n* = 8 (p# 20–29), HPAC *n* = 7 (p# 18–27), PaCaDD119 *n* = 8 (p# 2–18), 293T *n* = 10 (p# 11–21), TB32047 *n* = 8 (p# 19–27), 3T3L1 *n* = 6 (p# 13–20). (**G**) The effects of EPS-conditioned human myotube medium on total (per mL × 10^4^) and apoptotic/dead Panc1 cell numbers were measured. Mean (SD) of 9 independent experiments. (**H**) Relative cell death of Panc1 and PaCaDD119 after incubation with EPS-conditioned human myotube medium for 24–48 h was determined; mean (SD), number of independent experiments and passage number: Panc1 *n* = 16 (p# 2–13), PaCaDD119 *n* = 6 (p# 11–19). * *p* < 0.05, ** *p* < 0.01, *** *p* < 0.001; paired two-tailed *t*-test and Student’s *t*-test in (**A**,**B**,**E**); Student’s *t*-test in (**F**,**H**); Wilcoxon signed-rank and Mann–Whitney test in (**C**,**D**,**G**).

90% of PC patients are diagnosed with a pancreatic ductal adenocarcinoma, a very lethal form of cancer where effective therapies are lacking [28]. We stimulated the human pancreatic ductal adenocarcinoma cell lines Panc1 and HPAC, and the murine pancreatic cancer cell line TB32047 with pre- and post-intervention serum (collected after 12 weeks) of advanced PC patients with (*n* = 6) or without WB-EMS-mediated resistance exercise (*n* = 6) for 96 h. PC cell growth was significantly inhibited by the exercise serum pool, while non-malignant cells were unaffected (Figure 3A: Panc1, −8.53%, *p* = 0.027 (Ex pre 0.998 (mean) ± 0.115 (SD), Ex post 0.913 ± 0.129, Ctl pre 1.00 ± 0.133, Ctl post 0.982 ± 0.064); HPAC, −8.83%, *p* = 0.018 (Ex pre 0.89 ± 0.023, Ex post 0.811 ± 0.017, Ctl pre 1.00 ± 0.284, Ctl post 0.988 ± 0.099; TB32047, −10.33%, *p* = 0.0113 (Ex pre 1.101 ± 0.108, Ex post 0.902 ± 0.114, Ctl pre 1.00 ± 0.182, Ctl post 0.988 ± 0.092; Figure 3C: Panc1, −18.56%, *p* = 0.0078 (Ex pre 48.5 ± 5.95, Ex post 39.5 ± 7.16, Ctl pre 44.38 ± 5.5, Ctl post 48.19 ± 6.97; for additional statistical analysis, please refer to the legend of Figure 3). The anti-tumour effects of serum from exercising patients occurred in both, human and murine PC cells. A patient serum pool (*n* = 4) collected mid-exercise-intervention (after 6 weeks) also significantly decreased PC cell proliferation (Figure 3A, grey circles/black squares, Panc1, −5.72%, *p* = 0.0214 (Ex pre 0.985 ± 0.055, Ex post 0.929 ± 0.062, Ctl pre 1.00 ± 0.095, Ctl post 1.00 ± 0.071), indicating that the anti-cancer impact of resistance exercise using WB-EMS became effective already after a shorter training period. Furthermore, the incubation of PC cells with exercise serum pools from patients with other advanced-stage carcinomas, e.g., of the stomach, the colon/rectum or the prostate also significantly attenuated PC cell growth (Figure 3B; gastric, *n* = 6, −7.42%, *p* = 0.0153 (Ex pre 0.959 ± 0.08, Ex post 0.888 ± 0.087, Ctl pre 1.00 ± 0.063, Ctl post 1.03 ± 0.07); colorectal, *n* = 6, −16.6%, *p* = 0.0247 (Ex pre 0.842 ± 0.064, Ex post 0.702 ± 0.126, Ctl pre 1.00 ± 0.181, Ctl post 1.03 ± 0.059); prostate, *n* = 8, −11.23%, *p* = 0.0498 (Ex pre 0.884 ± 0.156, Ex post 0.785 ± 0.202, Ctl pre 1.00 ± 0.126, Ctl post 0.974 ± 0.048); for additional statistical analysis, please refer to the legend of Figure 3; for patient characteristics please refer to the Supplemental Table S1 and Schwappacher et al. [21]). These findings imply a general anti-tumour effect of exercise-conditioned serum, independent from the tumour entity in the donor patients. Importantly, neither human nor murine non-malignant cells were influenced by the exercise serum (Figure 3A,B; for statistical data, please refer to the legend of Figure 3). We could further demonstrate that PC cells undergo increased cell death when incubated with exercise-trained patient serum (Figure 3D, trypan blue exclusion, +71.36%, *p* = 0.0313 (Ex pre 5.89 ± 3.91%, Ex post 10.09 ± 4.51%, Ctl pre 6.69 ± 4.86%, Ctl post 5.53 ± 2.81%); Figure 3E, DNA fragmentation, +30.81%, *p* = 0.0667 (Ex pre 0.868 ± 0.151, Ex post 1.136 ± 0.132, Ctl pre 1.00 ± 0.251, Ctl post 0.701 ± 0.221)). This is an important finding as deregulation of apoptosis is a general mechanism of pancreatic ductal adenocarcinoma cells to ensure their own survival.

Electric pulse stimulation (EPS) of cultured skeletal muscle cells at the myotube stage can be used as an in vitro model of exercise [29,30]. Consistent with the observed effects of patient serum after WB-EMS-mediated resistance exercise, we found that EPS-conditioned medium from human or murine myotubes decreased proliferation of human and murine PC cells (Panc1, −6.91 ± 4.83% *p* = 0.0401; HPAC, −9.03 ± 6.1%, *p* = 0.0384; PaCaDD119, −12.16 ± 8.62%, *p* = 0.0424; TB32047, −11.48 ± 6.34%, *p* = 0.0002), while growth of non-malignant cells was not inhibited (293T, +5.54 ± 10.19%; 3T3L1, +5.5 ± 5.52; Figure 3F). The number of PC cells was also significantly reduced upon incubation with EPS medium (Panc1, −14.35%, *p* = 0.0171 (untreated 24.0 ± 2.81, 2× 20 min 20.56 ± 2.64), Figure 3G, left bars), while cell death, quantified by apoptotic/dead cell numbers (Panc1, +53.59%, *p* = 0.042 (untreated 15.31 ± 6.44%, 2× 20 min 23.52 ± 8.65%); Figure 3G, right bars) and DNA fragmentation (Panc1, +14.35%, *p* = 0.0408 (untreated 1.00 ± 0.146, 2× 20 min 1.143 ± 0.269); PaCaDD119, +29.09%, *p* = 0.0065 (untreated 1.00 ± 0.167, 2× 20 min 1.291 ± 0.208); Figure 3H), was increased. Taken together, these results further substantiate our previous conclusion that resistance exercise induces defensive humoral factors in patients with advanced cancer disease that target malignant cell growth and viability, while non-tumour cells are unaffected.

### 3.3. Exercise-Induced Myokines Control PC Cell Proliferation

**Figure 4:**
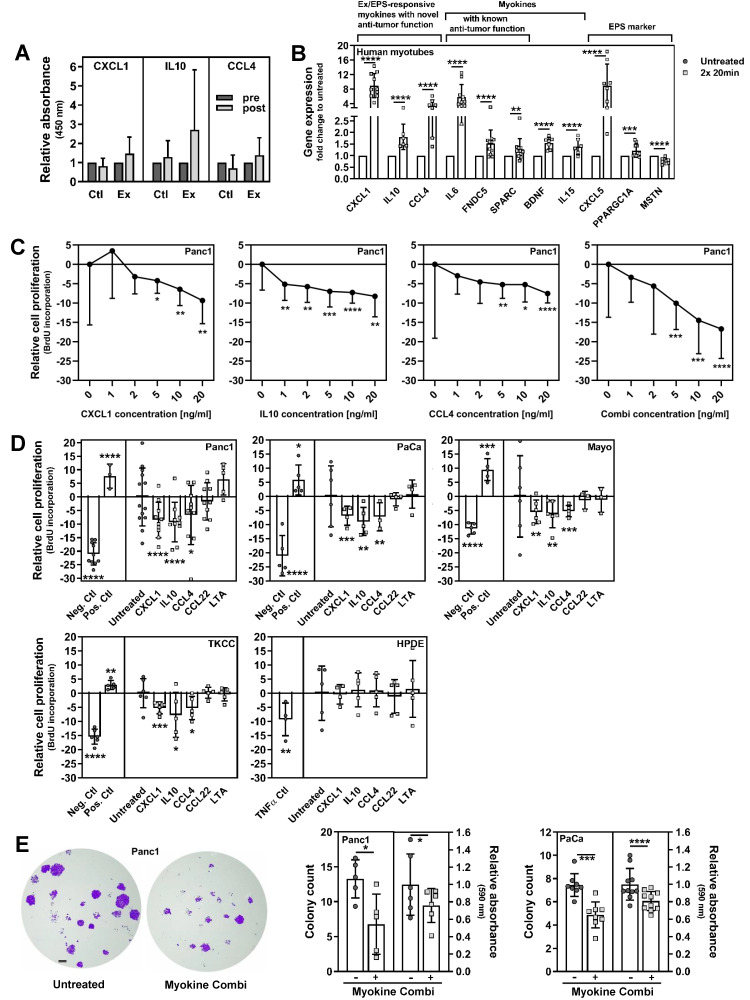
A set of exercise-induced myokines regulates growth behaviour of PC cells. (**A**) ELISA-based measurements of CXCL1, IL10 and CCL4 blood levels using plasma from control and exercise-trained patients with advanced PC. Absorbance values are shown relative to the pre-intervention plasma in both patient groups. For some patients a measurement was not possible due to detection sensitivity: CXCL1, control patients 5 out of 6 (5/6) and exercising patients 4/6; IL10, control patients 6/6 and exercising patients 6/6; CCL4, control patients 5/6 and exercising patients 3/6. Mean (SD) of 2 independent experiments. (**B**) Gene expression analysis in EPS-treated human myotubes shows the induction of CXCL1, IL10 and CCL4 as well as of established myokines (IL6, FNDC5/irisin, SPARC, BDNF and IL15). Known EPS marker genes (CXCL5, PPARGC1A and MSTN) are regulated accordingly. The graph depicts the fold changes relative to the values from untreated myotubes. Mean (SD); number of independent experiments: CXCL1 *n* = 10, IL10 *n* = 6, CCL4 *n* = 7, IL6 *n* = 10, FNDC5 *n* = 11, SPARC *n* = 12, BDNF *n* = 7, IL15 *n* = 6, CXCL5 *n* = 8, PPARGC1A *n* = 10, MSTN *n* = 10; passage number 2–6. (**C**) Panc1 cells were serum-starved and stimulated with increasing concentrations (1, 2, 5, 10 or 20 ng/mL) of recombinant human CXCL1 (left), human IL10 (middle left), human CCL4 (middle right) or with a combination of them (right) for 24 h. Cell proliferation was measured and is shown relative to the values after treatment without a recombinant protein (set as 0%). Mean (SD), number of independent experiments: CXCL1 *n* = 6, IL10 *n* = 7, CCL4 *n* = 6, combi *n* = 9; passage number 2–17. Additional statistics: CXCL1: 5 ng/mL *p* = 0.0105, 10 ng/mL *p* = 0.0038; IL10: 1 ng/mL *p* = 0.0075, 2 ng/mL *p* = 0.0033, 5 ng/mL *p* = 0.0008, 10 ng/mL *p* < 0.0001; CCL4: 5 ng/mL *p* = 0.0053, 10 ng/mL *p* = 0.0182; combi: 5 ng/mL *p* = 0.0004, 10 ng/mL *p* = 0.0001. (**D**) Different PC cells (Panc1, PaCaDD119, Mayo4636, TKCC10) and non-cancer pancreatic cells (HPDE) were serum-starved (except of HPDE) and treated with 20 ng/mL of CXCL1, IL10, CCL4, CCL22 or LTA for 24–48 h. Then, BrdU uptake was measured. The graphs depict cell proliferation relative to untreated cells (set as 0%). Prolonged starvation (or the addition of 20 ng/mL tumour necrosis factor α for HPDE) and normal growth conditions were used as negative and positive proliferation control, respectively. Mean (SD), number of independent experiments and passage number (p#): Panc1, CXCL1 *n* = 10, IL10 *n* = 10, CCL4 *n* = 10, CCL22 *n* = 10, LTA *n* = 5, p# 5–18; PaCaDD119, CXCL1 *n* = 4, IL10 *n* = 5, CCL4 *n* = 3, CCL22 *n* = 4, LTA *n* = 4, p# 3–8; Mayo4636, CXCL1 *n* = 6, IL10 *n* = 6, CCL4 *n* = 5, CCL22 *n* = 3, LTA *n* = 3, p# 6–18; TKCC10, CXCL1 *n* = 6, IL10 *n* = 5, CCL4 *n* = 5, CCL22 *n* = 5, LTA *n* = 5, p# 3–13; HPDE, CXCL1 *n* = 4, IL10 *n* = 5, CCL4 *n* = 5, CCL22 *n* = 5, LTA *n* = 5, p# 7–19. Additional statistics: Panc1: CCL22 *p* = 0.3509, LTA *p* = 0.1937; PaCaDD119: CCL22 *p* = 0.2874, LTA *p* = 0.6950; Mayo4636: CCL22 *p* = 0.2834, LTA *p* = 0.4724; TKCC10: CCL22 *p* = 0.8115, LTA *p* = 0.6868; HPDE: CXCL1 *p* = 0.7823, IL10 *p* = 0.6707, CCL4 *p* = 0.7119, CCL22 *p* = 0.6737, LTA *p* = 0.7499. (**E**) Panc1 and PaCaDD119 were stimulated with a combination of CXCL1, IL10 and CCL4 (20 ng/mL each) for 7–10 d, and colony formation was determined. After staining the cells with crystal violet, images were taken using the EZ4W stereo microscope with identical settings, and colonies were counted from random sections of each well. Images were processed using Photoshop (Adobe; sizing, contrast and brightness adjustment with identical settings for image acquisition of all samples in a given experiment); scale bar, 500 µm. Additionally, the stain was extracted, and the OD was measured (590 nm). Mean (SD), number of independent experiments and passage number: Panc1 count *n* = 6, absorbance *n* = 6, p# 2–12; PaCaDD119 count *n* = 8, absorbance *n* = 10, p# 11–18. * *p* < 0.05, ** *p* < 0.01, *** *p* < 0.001, **** *p* < 0.0001; Student’s *t*-test in B–D; Student’s *t*-test and Mann–Whitney test in (**E**).

These data encouraged us to analyse the blood of exercise-trained cancer patients in order to identify muscle-secreted factors, which might, at least partially, be responsible for the anti-proliferative and pro-apoptotic impact of exercise-conditioned serum on cancer cells. To determine myokines that are induced by resistance exercise using WB-EMS, we performed a cytokine array with plasma pools from our advanced PC patients. The plasma protein amount of the following six cytokines/myokines was substantially enhanced by the 12-week exercise training (Table 2 and Supplemental Table S2): C-X-C motif ligand 1 (CXCL1; ratio post-intervention/pre-intervention 1.88), interleukin 10 (IL10; 1.52), C-C motif chemokine ligand 4 (CCL4; 1.30), CCL22 (1.37), lymphotoxin α (LTA; 1.23), and angiogenin (1.21). Due to its well-known angiogenic character on cancer cells, we excluded angiogenin from our current research. In addition to increased myokine expression of e.g., IL6, brain-derived neutrophic factor (BDNF), and vascular endothelial growth factor (VEGF), as previously described [21], CXCL1, IL10, and CCL4 protein levels were also upregulated in the medium of human primary myotubes after EPS treatment (Table 3 and Supplemental Table S3; CXCL1 (ratio EPS-treated/untreated 3.36), IL10 (1.43), CCL4 (1.19)). However, we could not detect an increase in CCL22 and LTA protein amount in the myotube media as found in patient plasma after exercise, pointing towards either a non-muscle origin of these two protein factors, or an exercise-independent regulation. Since we investigated a plasma pool, patient-specific factors, such as medication, might be responsible for the induction of CCL22 and LTA in the exercise-trained serum pool. Apart from that, our results strongly suggest that CXCL1, IL10 and CCL4 are muscle-derived factors and that their expression and/or their release from skeletal muscle are enhanced by WB-EMS-mediated resistance training. We could also confirm a higher concentration of CXCL1, IL10 and CCL4 proteins in patient plasma after exercise via ELISA assays (Figure 4A). According to the literature, CXCL1, IL10 and CCL4 are more or less well described myokines, but as muscle-derived factors no significant anti-cancer effect has been attributed to them yet [2,30,31,32,33].

To consolidate our protein data from the cytokine arrays and the ELISA measurements, we performed gene expression experiments in human primary myotubes after in vitro exercise mediated by EPS. We found that the mRNA amount of CXCL1, IL10 and CCL4 was increased upon EPS (CXCL1, 8.95-fold ± 3.35, *p* < 0.0001; IL10, 1.81 fold ± 0.55, *p* < 0.0001; CCL4, 3.42 fold ± 1.64, *p* < 0.0001; Figure 4B). To strengthen our exercise-mimicking EPS approach, we included the expression analysis of known EPS-responsive markers and established myokines. CXCL5 (8.91 fold ± 5.94, *p* < 0.0001), PPARGC1A (peroxisome proliferator-activated receptor γ coactivator 1α; 1.22 fold ± 0.28, *p* = 0.0003) and MSTN (Myostatin; 0.76 fold ± 0.11, *p* < 0.0001) are regulated in muscle cells by EPS, confirming our previous results and data from the literature [21,34,35,36,37,38,39,40]. The myokines IL6 (5.8 fold ± 3.47, *p* < 0.0001), FNDC5 (1.52 fold ± 0.58, *p* < 0.0001), SPARC (1.26 fold ± 0.46, *p* = 0.0053), BDNF (1.54 fold ± 0.25, *p* < 0.0001) and IL15 (1.39 fold ± 0.34, *p* < 0.0001) are also induced by our EPS protocol [30,31,32,35,40,41,42].

Since our data from the cytokine arrays and the gene expression analysis strongly suggest that CXCL1, IL10 and CCL4 are induced in and released from skeletal muscle in PC patients after resistance exercise, we checked their impact on PC cell growth and viability. Dose response curves in Panc1 cells showed that recombinant human CXCL1, IL10 and CCL4 exert a significant anti-proliferative effect already at concentrations between 1 to 5 ng/mL, with the strongest effect at 20 ng/mL (CXCL1, −9.36 ± 5.97%, *p* = 0.0037; IL10, −8.26 ± 5.32%, *p* = 0.0017; CCL4, −7.56 ± 2.41%, *p* < 0.0001; Figure 4C). The combination of the three myokines showed a synergistic effect with a suppression of Panc1 proliferation of more than 16% upon the incubation with 20 ng/mL of each protein (−16.72 ± 7.60%, *p* < 0.001; Figure 4C, right graph; for additional statistical analysis, please refer to the legend of Figure 4). Furthermore, we could show that the stimulation of different PC cells with 20 ng/mL of CXCL1, IL10 or CCL4 significantly decreased their proliferation, while the non-malignant pancreatic cells HPDE were unaffected (CXCL1: Panc1 −8.50 ± 6.6%, *p* < 0.0001; PaCaDD119 −6.86 ± 2.92%, *p* = 0.0009; Mayo4636 −5.52 ± 4.2%, *p* = 0.0092; TKCC10 −5.25 ± 2.1%, *p* < 0.0001; IL10: Panc1 −9.27 ± 7.32%, *p* < 0.0001; PaCaDD119 −8.94 ± 4.48%, *p* = 0.0017; Mayo4636 −6.31 ± 4.8%, *p* = 0.0091; TKCC10 −7.73 ± 7.97%, *p* = 0.0396; CCL4: Panc1 −6.69 ± 10.87%, *p* = 0.0245; PaCaDD119 −7.21 ± 4.08%, *p* = 0.0066; Mayo4636 −5.19 ± 2.02%, *p* < 0.0001; TKCC10 −5.24 ± 4.14%, *p* = 0.012; Figure 4D). As additional controls, we examined the effect of recombinant human CCL22 and LTA, proteins which were increased in the plasma of PC patients doing exercise but not in the EPS myotube medium (please see Table 2 and Table 3), but found no significant suppressive effect on PC cell growth (Figure 4D). We also tested the impact of CXCL1/IL10/CCL4 on the colony forming ability of PC cells (Figure 4E); this in vitro technique examines the capability of tumour cells to form large colonies. We found that PC colony formation was significantly suppressed by the myokine mix (Panc1: counting −49% (untreated 13.26 ± 2.74, Myokine combi 6.77 ± 4.3), *p* = 0.0317; OD −24.17% (untreated 1.00 ± 0.35, Myokine combi 0.76 ± 0.2), *p* = 0.013; PaCaDD119: counting −34.83% (untreated 7.45 ± 0.99, Myokine combi 4.85 ± 1.12), *p* = 0.0003; OD −19.18% (untreated 1.00 ± 0.19, Myokine combi 0.81 ± 0.11), *p* < 0.0001; Figure 4E), further consolidating our hypothesis that muscle-derived CXCL1, IL10 and CCL4 are regulators of PC cell growth.

### 3.4. Myokine-Mediated Regulation of PC Cell Migration

**Figure 5:**
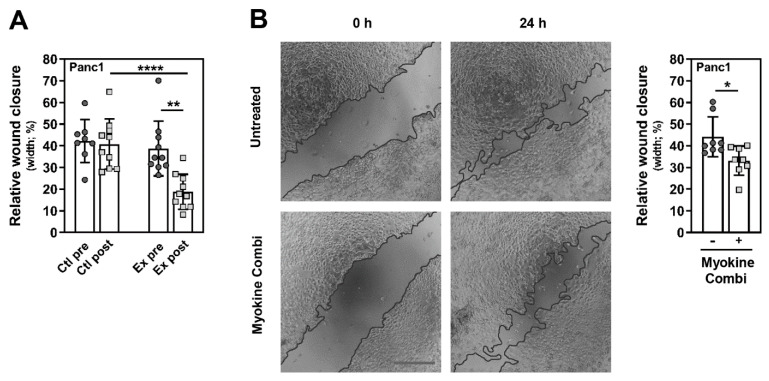
Exercise-conditioned serum from advanced PC patients and the exercise-induced myokines CXCL1, IL10 and CCL4 reduce migration of PC cells. (**A**,**B**) Panc1 cells were serum-starved, scratch-wounded and then stimulated with either 10% serum from exercising or control patients with stage III/IV PC (**A**), or a combination of CXCL1, IL10 and CCL4 (20 ng/mL each; (**B**)) for 24 h. The migratory ability of the PC cells was measured by determining the percentage of wound closure at the same location relative to directly after scratching. Representative pictures for the myokine effect are shown in B (taken with the EVOS digital inverted microscope; the dark lines mark the wound edges (scale bar, 500 µm)). Mean (SD), number of independent experiments: serum *n* = 10, myokine combi *n* = 8, passage number 7–21. * *p* < 0.05, ** *p* < 0.01, **** *p* < 0.0001; Wilcoxon-signed rank and Mann–Whitney test.

In addition, we checked for effects of CXCL1, IL10 and CCL4 on the mobility of PC cells using the scratch wound healing assay. Firstly, Panc1 cells were treated with pre- or post-intervention serum from advanced PC patients for 24 h. As shown in Figure 5A, the wound closure over the scratch of Panc1 cells was significantly reduced after stimulation with exercise-conditioned serum compared to pre-intervention serum from the exercise group and serum from the controls (Ctl: pre 42.14 ± 9.94%, post 40.83 ± 11.53%; Ex: pre 38.78 ± 12.66%, post 18.82 ± 8.06%; Ex post vs. pre *p* = 0.0020; Ctl post vs. Ex post *p* < 0.0001). When Panc1 cells were treated with a combination of CXCL1, IL10 and CCL4 for 24 h, the wound healing over the scratch was also significantly decreased (Figure 5B). The analysis revealed that the wound of untreated Panc1 cells has closed by 44.10 ± 9.19% in contrast to myokine-stimulated cells, where only 33.09 ± 6.75% of the initial scratch area was covered by cells (*p* = 0.0207; Figure 5B). The results suggest that CXCL1, IL10 and CCL4, which were induced by WB-EMS resistance exercise, decreased the mobility of PC cells.

### 3.5. CXCL1, IL10 and CCL4 Induce PC Cell Death

**Figure 6:**
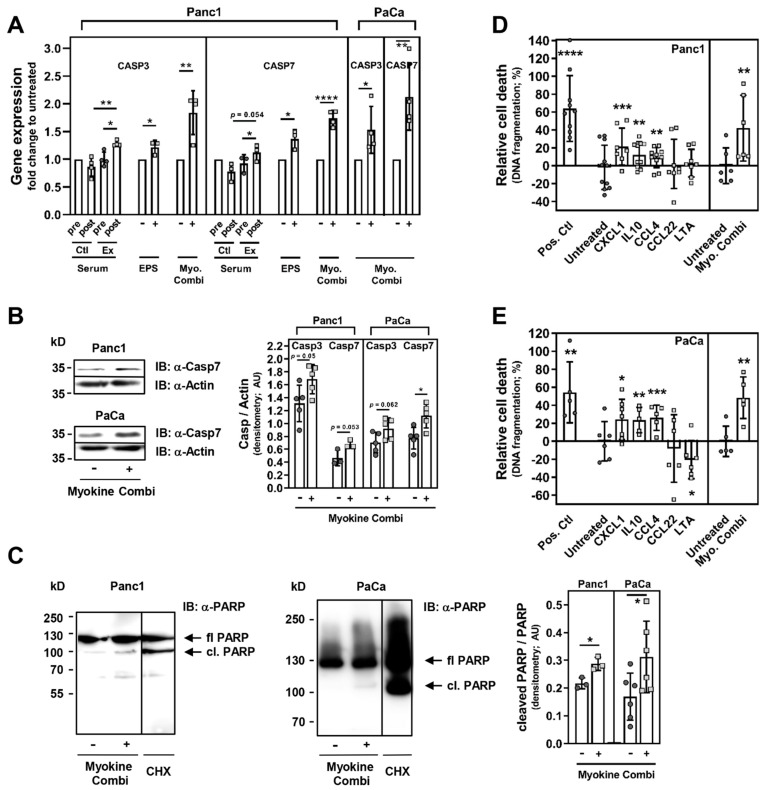
Apoptotic events are regulated by CXCL1, IL10 and CCL4 in PC cells. (**A**) Panc1 and PaCaDD119 cells were serum-starved and stimulated either with control or exercise-conditioned serum from advanced PC patients, with an EPS-conditioned medium pool from human myotubes, or with a combination of CXCL1, IL10 and CCL4 (20 ng/mL each) for 48–72 h. Then, CASP3 and 7 mRNA expression levels were analysed, and results are depicted in the graph as fold changes to the respective control/untreated sample. Mean (SD), number of independent experiments and passage number (p#): Panc1, CASP3, serum *n* = 4, EPS *n* = 3, Myokine combi *n* = 4, p# 7–15; Panc1, CASP7, serum *n* = 3, EPS *n* = 3, Myokine combi *n* = 4, p# 8–17; PaCaDD119, CASP3, Myokine combi *n* = 4, p# 5–15; PaCaDD119, CASP7, Myokine combi *n* = 4, p# 7–16. (**B**,**C**) Immunoblot analysis of caspase 3 and 7 expression and PARP cleavage after treatment with myokines. As described in Figure 6A, Panc1 and PaCaDD119 were serum-deprived and stimulated with a combination of CXCL1, IL10 and CCL4. As a control for cell death-associated PARP cleavage, some cells were treated with 100 µg/mL cycloheximide (CHX) for 4–6 h before lysis. Cell lysates were analysed using either Casp7, Casp3 (data shown only in the graph) and α-Actin, or PARP (full length (fl) and cleaved (cl, 89 kDa fragment)) immunoblotting. Signal intensities were quantified using ImageJ (including background and protein loading; mean (SD), graphs, number of independent experiments and passage number: Panc1, Casp3 *n* = 5, Casp7 *n* = 3, cl. PARP *n* = 3, p# 7–16; PaCaDD119, Casp3 *n* = 5, Casp7 *n* = 5, cl. PARP *n* = 6, p# 5–15). (**D**,**E**) PC cells were incubated with 20 ng/mL of CXCL1, IL10, CCL4, CCL22 or LTA, or with a combination of CXCL1/IL10/CCL4 for 24–48 h (Panc1) or 48–72 h (PaCaDD119). Then, relative cell death was determined by DNA fragmentation; mean (SD), number of independent experiments and passage number: Panc1, CXCL1 *n* = 7, IL10 *n* = 10, CCL4 *n* = 10, CCL22 *n* = 8, LTA *n* = 8, combi *n* = 6, p# 8–22; PaCaDD119, CXCL1 *n* = 7, IL10 *n* = 5, CCL4 *n* = 5, CCL22 *n* = 6, LTA *n* = 6, combi *n* = 5, p# 12–19. Additional statistics: Panc1: CCL22 *p* = 0.7577, LTA *p* = 0.4351; PaCaDD119: CCL22 *p* = 0.5770, LTA *p* = 0.0256. * *p* < 0.05, ** *p* < 0.01, *** *p* < 0.001, **** *p* < 0.0001; paired two-tailed *t*-test and Student’s *t*-test in A; Student’s *t*-test in (**B**–**E**).

In our previous study, a gene array from prostate cancer cells with cancer-associated genes revealed that specific genes controlling cancer cell growth and death, are regulated by resistance exercise using WB-EMS [21]. For the present report, we performed further experiments in PC cells. We determined that the mRNA expression of CASP3 and 7 was significantly upregulated after a 72 h treatment with exercise serum from advanced-stage PC patients (post- vs. pre-exercise serum: CASP3, 1.28 fold ± 0.05, *p* = 0.043; CASP 7, 1.12 fold ± 0.15, *p* = 0.0118; post-intervention serum exercise vs. control group: CASP3, *p* = 0.0034; CASP7, *p* = 0.0543, Figure 6A), as well as with EPS-conditioned myotube medium (CASP3, 1.22 fold ± 0.12, *p* = 0.0396; CASP 7, 1.37 fold ± 0.17, *p* = 0.0206). Importantly, PC cells treated with a combination of CXCL1/IL10/CCL4 for 48–72 h also showed a significantly higher amount of CASP3 and CASP7 mRNA compared to untreated cells (Panc1: CASP3, 1.84 fold ± 0.4, *p* = 0.0054; CASP 7, 1.74 fold ± 0.14, *p* < 0.0001; PaCaDD119: CASP3, 1.53 fold ± 0.42, *p* = 0.0486; CASP 7, 2.13 fold ± 0.6, *p* = 0.0095; Figure 6A). Using immunoblot analysis, we could determine a moderate, yet apparent increase in protein expression of caspase 3 (Panc1: +28.58% (untreated 1.31 ± 0.28, Myokine combi 1.69 ± 0.23), *p* = 0.0502; PaCaDD119, +31.3% (untreated 0.70 ± 0.16, Myokine combi 0.92 ± 0.16), *p* = 0.0624; immunoblot not shown) and caspase 7 (Panc1: +45.52% (untreated 0.47 ± 0.12, Myokine combi 0.68 ± 0.06), *p* = 0.0531; PaCaDD119, +45.42% (untreated 0.77 ± 0.17, Myokine combi 1.12 ± 0.19), *p* = 0.0144; Figure 6B). Furthermore, we performed immunoblot analysis for the cleavage of the caspase 3/7 substrate PARP on PC cell lysates after incubation with CXCL1/IL10/CCL4 for 48–72 h (Figure 6C). The expression analysis showed a significant increase in PARP cleavage (Panc1: +32.4% (untreated 0.22 ± 0.02, Myokine combi 0.29 ± 0.02), *p* = 0.0172; PaCaDD119: +54.07% (untreated 0.17 ± 0.09, Myokine combi 0.31 ± 0.13), *p* = 0.0462; Figure 6C). Subsequent DNA fragmentation analysis revealed that CXCL1, IL10 and CCL4 alone and, in a synergistic manner, in combination induces the death of PC cells (Panc1: CXCL1, +21.44 ± 20.71%, *p* = 0.0001; IL10, +12.20 ± 14.68%, *p* = 0.0014; CCL4, +9.15 ± 11.2%, *p* = 0.0016; combination, +42.04 ± 36.85%, *p* = 0.0067; Figure 6D; PaCaDD119: CXCL1, +24.23 ± 22.45%, *p* = 0.0145; IL10, +23.67 ± 14.04%, *p* = 0.0011; CCL4, +26.14 ± 14.05%, *p* = 0.0005; combination, +48.48 ± 23.17%, *p* = 0.0016; Figure 6E). Treatment with CCL22 or LTA served as additional controls and had no stimulatory effect on PC cell death (Figure 6D,E). Taken together, our data show a myokine-induced upregulation of caspase 3 and 7 expression and PARP cleavage, and an increase in PC cell apoptosis and death upon treatment with CXCL1/IL10/CCL4.

## 4. Discussion

In the study presented here, we investigated the direct effect of resistance training in advanced aggressive tumour disease on cancer cell lines, in order to gain more knowledge on the cross-talk between skeletal muscle and tumour.

**Figure 7:**
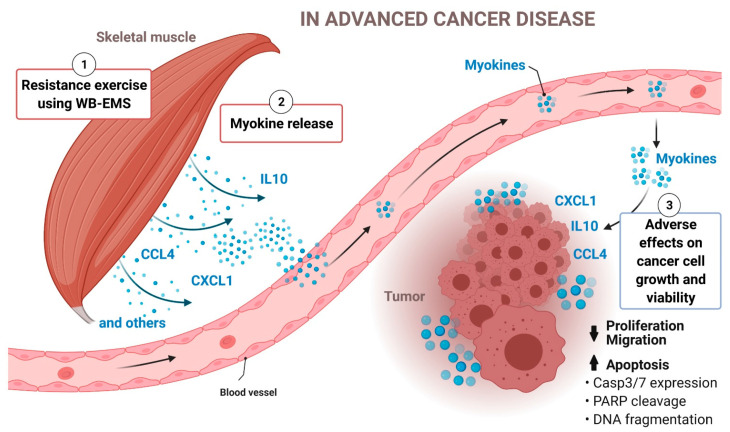
The model depicts a mechanistic linkage between muscle activation by WB-EMS-mediated resistance exercise and cancer cell death in advanced cancer disease. The muscle-derived factors IL10, CXCL1 and CCL4 are, at least in part, responsible for the exercise-mediated upregulation of caspase 3 and 7 expression in cancer cells. This correlates with the enhanced cleavage of the caspase 3/7 substrate PARP. Once cleaved, PARP cannot repair DNA damage, leading to more DNA fragmentation and ultimately to increased cancer cell death (generated using Biorender.com, accessed on 22 July 2021; we confirm the copyright of the image).

The exercise-conditioned serum was derived from advanced-stage cancer patients, with focus on patients with PC, who participated in our pilot study and completed a 12-week WB-EMS training, a form of resistance exercise feasible for physically weakened cancer patients [20]. We found that the exercise-trained patient serum inhibits the growth and migration of PC cells, and induces PC cell death. Besides other factors, we identified higher levels of IL10, CXCL1 and CCL4 in the blood of exercising PC patients. Together with data from an EPS-based in vitro exercise model using human skeletal muscle cells, we propose that IL10, CXCL1 and CCL4 are muscle-derived myokines that are induced by WB-EMS-mediated resistance exercise. We found that single administration of recombinant IL10, CXCL1 or CCL4 attenuates the proliferation of PC cells, and simultaneously induces pro-apoptotic effects, while the stimulation of these cells with a combination of CXCL1/IL10/CCL4 synergistically increased the observed anti-tumour effects. Furthermore, our results point towards a function of caspase 3 and 7 in exercise-induced apoptosis of cancer cells and further corroborate the existence of a mechanistic linkage between muscle activity and cancer cell death. We propose that muscle-derived IL10, CXCL1 and CCL4 are, at least in part, responsible for the exercise-mediated upregulation of CASP3 and 7 gene expression and consequently their increased protein levels in PC and other cancer cells. This seems to correlate with enhanced cleavage of PARP, which is an essential step in apoptotic events and depends on caspase 3/7 activity. Once cleaved, PARP cannot repair DNA damage, causing DNA fragmentation and ultimately cancer cell death (please see Figure 7; [43,44]).

The majority of studies, which investigated the effects of exercise-conditioned serum on cancer, focused either on healthy individuals, patients in an early stage of cancer disease or cancer survivors [45,46,47]. We here present evidence that physically weakened patients with advanced cancer are able to effectively exercise in order to secret muscle-derived factors with anti-cancer effects. Taken together with the data from our previous report [21], we show that serum from exercise-trained patients with aggressive gastrointestinal (GI) cancer (pancreas, stomach), with less aggressive GI tumours (colon/rectum) or non-GI tumours (prostate) reduce growth and viability of various cancer cells. Our results strongly suggest that resistance exercise using WB-EMS triggers an anti-tumour response from skeletal muscle that is independent from the tumour entity. Yet, the limited number of exercise-trained patients may have affected our promising results. Also, the heterogeneity of our study population (sex, age, tumour biology) might have influenced the outcome. To strengthen our observations, a larger and more homogeneous population of patients should be studied in future research. In our report we focused on the anti-cancer effects of exercise on tumour cells; however, a pancreatic ductal adenocarcinoma is composed of various cell types, e.g., cancer-associated fibroblasts. It will be very important to investigate the impact of WB-EMS-mediated resistance exercise and myokines on other cells of the tumour microenvironment.

The aetiology of PC has been extensively analysed. Ninety percent of the PC cases are diagnosed with a pancreatic ductal adenocarcinoma, which is highly fatal with currently limited options for early detection and effective treatment [28]. There is moderate evidence for an association between higher levels of physical activity and lower occurrence of PC [28,48]. In recent years, several clinical trials have been conducted with an exercise intervention in PC patients, focusing on the effects of exercise on physical function and the psychological condition of the patients [49,50]. However, knowledge about the more direct effects of exercise to fight this type of malignant tumour disease is scarce. As mentioned, skeletal muscle releases myokines upon contraction, which allow communication with various body tissues and the muscle itself. Hundreds of myokines, including interleukins like IL6 and IL15, and growth factors like BDNF and VEGF, are known, but so far only for a few myokines a biological function is described [4,6]. Hojman and colleagues reported for the first time, that conditioned serum from exercising mice decreases growth and viability of breast cancer cells via exercise-induced factors. Further examination revealed that the myokine oncostatin M is involved in exercise-induced apoptosis in breast cancer cells [2]. The Hojman group also reported an IL6-dependent mechanism that activates the innate immune defense upon exercise to control tumour growth [11]. SPARC (secreted protein acidic and rich in cysteine) was also released from skeletal muscle upon contraction and suppressed colon tumourigenesis in exercising mice, primarily via the induction of apoptosis in colon carcinoma cells [12]. Furthermore, several in vitro studies described FNDC5/irisin as a myokine with diverse anti-tumour activities in various cancer cells [15,16,17,18,51,52,53,54]. Gannon and co-workers demonstrated that irisin suppresses growth and migration of breast cancer cells [51]. In lung and bone cancer cells it was shown that irisin inhibits cell proliferation, migration and invasion, and affects epithelial-to-mesenchymal transition (EMT) [18,53]. Liu et al. reported that irisin attenuates migration and invasion of PC cells through inhibition of EMT, and suppresses PC cell growth via AMPK/mTOR signalling [16]. It is important to further examine the anti-migratory effect of serum after WB-EMS exercise and of the myokines CXCL1, IL10 and CCL4, we have seen in our experiments. Since the inhibitory effect of exercise serum on PC cell migration was stronger than with myokine supplementation, other exercise-induced factors, like irisin and its regulation of EMT, might also play a role in the exercise-mediated regulation of PC mobility. We demonstrated in our previous study that WB-EMS-mediated resistance exercise regulates cancer cell growth and viability via the control of exercise-sensitive genes encoding proteins for cell cycle control, cell growth signalling and the regulation of apoptosis [21]. It is possible that resistance exercise initiates epigenetic modification, involving e.g., post-translational modifications of histone proteins or expression of microRNAs [21,55]. Various studies have shown that physical activity/exercise can induce epigenetic modification in cells of the tumour microenvironment, in natural killer cells and directly in cancer cells, suggesting that epigenetic changes play a significant role in cancer initiation, progression and invasion [55]. Future studies should give mechanistic insight into how WB-EMS-mediated resistance exercise and thereby induced myokines affect gene expression in cancer cells.

Our data presented here suggest that resistance exercise using WB-EMS induces the release of IL10, CXCL1 and CCL4 from skeletal muscle in patients with advanced PC. In the literature, these cytokines are described as myokines [2,30,31,32]. IL10 was, among others, identified as an exercise-induced muscle factor with a minor anti-proliferative effect on breast cancer cells [2]. Secreted by various immune cells, IL10 participates in the suppression of cancer-mediated inflammation. Its role in cancer is controversially discussed, but the majority of studies report anti-proliferative effects on tumour cells [56]. Secreted from activated skeletal muscle, IL10 is mostly described as an anti-inflammatory regulator with an indirect impact on cancer [57,58]. CXCL1, also named growth-related oncogene 1 alpha (GRO1α), is an established myokine [30,59]. It plays a major role in inflammation, angiogenesis, tumourigenesis, and wound healing, and is considered as a prognostic factor in cancer, but the data are controversial [60,61]. The majority of reports suggest that CXCL1 promotes tumourigenesis in various cancers. However, we clearly show (dose-dependent) anti-proliferative and pro-apoptotic effects of recombinant CXCL1 on several PC cell lines, which indicates cancer-protective properties of CXCL1 within our experimental approach. When induced by contraction in skeletal muscle, CXCL1 regulates muscle cell differentiation and motility, as well as fatty acid oxidation in the muscle [33,34]. To our knowledge, a direct role of muscle-derived CXCL1 on cancer cells has not been reported yet. CCL4, also referred to as macrophage inflammatory protein 1 beta (Mip1β), was described as an exercise-triggered muscle factor [62], but controversial data can be found in the literature [63,64,65]. CCL4 is known to be chemoattractant for various immune cells. There is evidence that CCL4 indirectly promotes cancer development and progression by recruiting certain immune cells and by influencing cells of the tumour microenvironment to enhance their pro-tumourigenic capacities [66]. As a myokine, CCL4 participates in the control of myoblast growth and in wound repair after muscle injury [67,68]. Muscle-derived CCL4 has not been attributed with direct anti-tumour effects yet. In our previous report, we suggested that the anti-proliferative and pro-apoptotic effects of exercise on cancer cells—transmitted via exercise- or EPS-conditioned serum/medium—are caused by myokines. Based on our data presented here, we have defined three additional mediators of the anti-tumour effects of exercise. As mentioned before, the established myokines IL6, irisin and SPARC have cancer-protective functions [11,12,15,16,17,18,51,52,53,54]. Since we also found increased mRNA levels of these myokines in human myotubes after exercise-mimicking EPS, it is possible that IL6, irisin and/or SPARC also contribute to the anti-tumour effects of our exercise-conditioned patient serum and muscle cell medium. 

A major reason for the poor prognosis of patients with pancreatic duct carcinomas is their insensitivity to most anti-cancer therapeutic approaches. PC is a highly chemoresistant malignancy and most cytotoxic therapies aim to induce apoptosis in these cancer cells. Yet, pancreatic duct adenocarcinoma cells have developed multiple molecular alterations to escape apoptosis and ensure their own survival. They acquired resistance to apoptotic stimuli such as death ligands or anti-cancer drugs by diverse molecular mechanisms that either disrupt a pro-apoptotic signal or antagonise apoptosis [69,70]. We demonstrate here that the expression of caspase 3 and 7 is upregulated by exercise-conditioned serum from advanced PC patients, by myotube medium conditioned via in vitro exercise, as well as by a combination of the myokines CXCL1/IL10/CCL4 in different PC cells. Classically, apoptosis is initiated either via intracellular signals when cells are under stress (intrinsic pathway) or via extracellular signals activating death receptors on the cell surface (extrinsic pathway); both pathways ultimately activate the executioner caspases 3, 6 and 7 [69,71]. The upregulation of gene and protein expression of caspases 3 and 7, as shown here, might be an alternative route to induce apoptosis, whereby the classical initiation of apoptosis is bypassed: it is possible that the exercise-induced factors CXCL1, IL10 and CCL4 enhance cancer cell apoptosis by increasing the availability of caspase 3 and 7. Several reports showed a loss/downregulation of caspase 3 and/or 7 in cancer cells which correlated with the resistance to apoptosis [72,73,74,75]. We observed that higher expression levels of caspase 3 and 7 correlated with enhanced PARP cleavage in PC cells. The cleavage of PARP by caspase 3/7 separates PARP into 2 segments. The smaller fragment includes the DNA binding domain, which irreversibly binds to nicked DNA where it acts as inhibitor of active full length PARP as well as of other DNA repair enzymes; thereby, DNA repair is impeded [76]. We observed that DNA fragmentation in PC cells is enhanced after stimulation with myokines, which ultimately leads to increased PC cell death. Some oncological therapeutic regimens include PARP inhibitors as potentiators of chemotherapeutics, leading to malfunction of DNA repair mechanisms and thus to genomic dysfunction and cancer cell death [77].

Our data indicate that muscle-derived CXCL1, IL10 and CCL4 are part of the mechanistic link between exercise and the induction of apoptosis in cancer cells. Since there is discrepancy regarding the anti-cancer impact of CXCL1 and CCL4 between our results and some data from the literature, it is of interest whether the site of expression determines different biological functions of CXCL1 and CCL4. It is known that the prototypical myokine IL6 has a strong pro-inflammatory function when released by immune cells, while muscle-derived IL6 acts anti-inflammatory [78]. The opposite actions of IL6 seem to be due to a switch of IL6 signalling from a canonical mode, which occurs in myeloid cells, to a trans-signalling mode, which can be found in adipocytes and muscle cells; an increased expression of the ADAM10/17 metalloprotease promotes the trans-signalling via the soluble IL6 receptor α [4]. More research is necessary to investigate if similar mechanisms determine tumour-inhibitory vs. pro-tumour effects of CXCL1 and CCL4. Additionally, it will be important to examine whether the induction of this set of myokines after exercise is universal. Not much is known about how different types of exercise induce different sets of myokines. It needs to be clarified if CXCL1, IL10 and/or CCL4 are rather specifically released after resistance exercise or are also induced by endurance training. Furthermore, our data suggest that WB-EMS-mediated resistance exercise triggers a comparable anti-tumour response in patients with different GI and non-GI tumour diseases. So far, we have identified CXCL1, IL10 and CCL4 in exercise-conditioned serum of advanced PC patients; it will be important to know if these myokines are generally induced by exercise, independent of the tumour type and its characteristics (e.g., aggressiveness or hormone dependency). The patients’ physical status might also affect myokine availability. For the well-characterised IL6, it is reported to increase exponentially during exercise and that the extent of the IL6 surge depends on the amount of activated skeletal muscle mass [79]. It was also shown that circulating levels and muscle levels differ for certain myokines in patients with cancer cachexia vs. non-cachectic cancer patients [80], indicating that the amount of muscle mass and/or the “quality” of muscle contraction is a significant parameter in the regulation of myokine release. Detailed knowledge about the expression conditions and dynamics of post-exercise secretory factors with anti-tumour function would be very important for cancer patients. Multimodal therapeutic concepts could then include highly personalised physical training programs with the best anti-cancer impact.

## 5. Conclusions

Regular physical exercise reduces the cancer risk, but the exercise-malignancy relationship is not fully understood. Several myokines, that are released by skeletal muscle, have direct anti-tumour function. We believe we have identified additional exercise-induced myokines with cancer-protective properties in patients with advanced PC. We could show that IL10, CXCL1 and CCL4 inhibit growth and migration of PC cells, and induce apoptotic events. The identification of myokines with anti-tumour function in patients with advanced-stage PC after exercise strongly supports sport therapies for cancer patients.

## Figures and Tables

**Table 1:** Pre-intervention characteristics of the PC patient cohort. Values are given as mean (SD). Where appropriate, statistical analysis was done using independent samples *t*-test (^a^) or Mann–Whitney test (^b^).

Parameter	Pancreatic Cancer (PC)		*p*
	Control (*n* = 6)	Resistance Exercise Using WB-EMS (*n* = 6)	
**Sex**			-
Male, *n* (%)	3 (50%)	4 (60%)	
Female, *n* (%)	3 (50%)	2 (40%)	
**Age** (y)	61.0 ± 8.4	62.7 ± 9.1	0.748 ^a^
**Tumor Stage** (UICC)			-
III, *n* (%)	1 (20%)	1 (20%)	
IV, *n* (%)	5 (80%)	5 (80%)	
**Oncological Therapy**			-
Chemotherapy, *n* (%)	6 (100%)	6 (100%)	
Other therapies, *n* (%)	-	-	
**Karnofsky Index** (%)	78.3 ± 4.1	83.3 ± 10.3	0.178 ^b^
**6 min-Walking Distance** (m)	557.5 ± 75.9	586.0 ± 54.5 (*n* = 5)	0.574 ^b^
**Body Parameters**			
Body Weight (kg)	74.6 ± 9.7	67.8 ± 6.9	0.193 ^a^
Weight Lossin the last 3–6 month (%)	3.9 ± 5.1	9.0 ± 11.6	0.708 ^b^
Body Mass Index (kg/m^2^)	25.6 ± 1.4	23.5 ± 2.5	0.093 ^a^
Skeletal Muscle Mass (kg)	23.9 ± 6.7	22.0 ± 4.4	0.582 ^a^
**Blood Parameters**			
Albumin (g/L)	42.5 ± 2.2	40.9 ± 2.1	0.226 ^a^
C-reactive Protein (mg/L)	19.7 ± 33.7	5.7 ± 3.3	0.335 ^a^
Creatinine (mg/dL)	0.8 ± 0.1	0.8 ± 0.2	0.606 ^a^
Hematocrit (%)	36.0 ± 2.1	37.8 ± 3.4	0.283 ^a^
Hemoglobin (g/dL)	12.2 ± 0.8	12.7 ± 1.1	0.434 ^a^
Leucocytes (×10^3^/µL)	5.2 ± 2.3	5.5 ± 3.3	0.851 ^b^
Erythrocytes (×10^6^/µL)	3.9 ± 0.2	4.2 ± 0.3	0.143 ^b^
Thrombocytes (×10^3^/µL)	234.5 ± 133.4	181.2 ± 69.5	0.818 ^b^

**Table 2:** Potential myokines in conditioned plasma from advanced-stage PC patients after WB-EMS-mediated resistance exercise. For simultaneous detection of cytokines/myokines in the pre- and post-intervention plasma pools of exercise-trained (*n* = 6) or non-exercising control patients (*n* = 6), a cytokine array was performed. For details regarding the determination of the presented changes, please refer to Material and Methods. Differences between the signal intensities of >20% were considered significant and the respective cytokines/myokines were subjected to further analysis.

Target	Control		Exercise		Ratio Post-Intervention	
	**pre**	**post**	**pre**	**post**	**Ctl**	**Ex**
**CXCL1 (Groα)**	1.00	1.05	1.00	1.88	1.00	1.59
**IL10**	1.00	0.69	1.00	1.52	1.00	1.86
**CCL4 (Mip1β)**	1.00	0.84	1.00	1.30	1.00	1.54
**CCL22 (MDC)**	1.00	0.97	1.00	1.37	1.00	1.47
**LTA (TNFβ)**	1.00	0.74	1.00	1.23	1.00	1.36

**Table 3:** Exercise-mimicking EPS induces CXCL1, IL10 and CCL4 in vitro. Primary human myotubes were treated with EPS (based on WB-EMS training parameters) and a serum pool from 3 independent EPS treatments was used for a cytokine array. The change in protein expression of the identified exercise-induced cytokines (please see Table 2) in EPS-conditioned vs. unconditioned myotube medium is shown.

Target	Untreated	2× 20 min EPS
**CXCL1 (Groα)**	1.00	3.36
**IL10**	1.00	1.43
**CCL4 (Mip1β)**	1.00	1.19
**CCL22 (MDC)**	1.00	0.86
**LTA (TNFβ)**	1.00	0.84

## Data Availability

Data sharing not applicable.

## Supplementary Materials

The following are available online at https://www.mdpi.com/article/10.3390/cancers13153820/s1, Table S1: Pre-intervention characteristics of the patient cohort with gastric cancer, Table S2: Cytokine Array with plasma from non-exercising control and exercising PC patients, Table S3: Cytokine Array with medium from human myotubes after EPS. File S1: original western blot images.

## Abbreviations

PC: pancreatic cancer; GI, gastrointestinal; WB-EMS, whole-body electromyostimulation; Ex, exercise; EPS, electric pulse stimulation; HSMMs, human skeletal muscle myoblasts; CXCL1, C-X-C motif ligand 1; CXCL5, C-X-C motif ligand 5; CCL4, C-C motif chemokine ligand 4; IL6, interleukin 6; IL10, interleukin 10; IL15, interleukin 15; BDNF, brain-derived neutrophic factor; CCL22, C-C motif chemokine ligand 22; LTA, lymphotoxin α; VEGF, vascular endothelial growth factor; SPARC, secreted protein acidic and rich in cysteine; FNDC5, fibronectin type III domain-containing protein 5; PPARGC1A, peroxisome proliferator-activated receptor γ coactivator 1α; MSTN, myostatin; CASP3, caspase 3; CASP7, caspase 7; PARP, poly(ADP-ribose) polymerase; EMT, epithelial-to-mesenchymal transition; BrdU, 5-bromo-2′-deoxyuridine incorporation; GAPDH, glyceraldehyde-3-phosphate dehydrogenase; HPRT1, hypoxanthine phosphoribosyltransferase 1; RPLP0, ribosomal protein lateral stalk subunit P0.
