# Supplementary material for: Muscle-Derived Cytokines Reduce Growth, Viability and Migratory Activity of Pancreatic Cancer Cells
Source: Cancers (Basel). 2021 Jul 29;13(15):3820. doi: 10.3390/cancers13153820 (PMC8345221; doi:10.3390/cancers13153820)
Supplement: Supplementary file 1 [file cancers-13-03820-s001.zip › cancers-1278687-original images.pdf]

Figure 6B Panc1

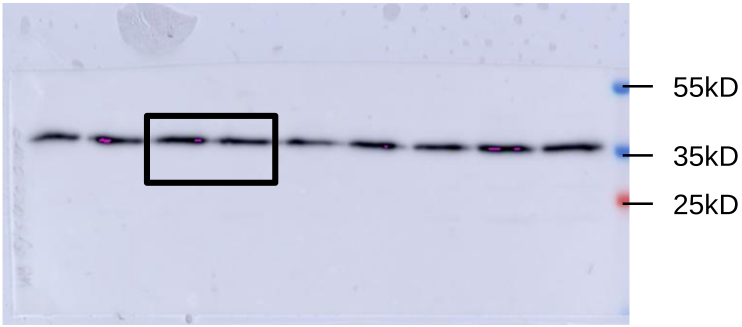

JPEG
anti-Casp7 (RRID:AB_2687912)
with protein marker

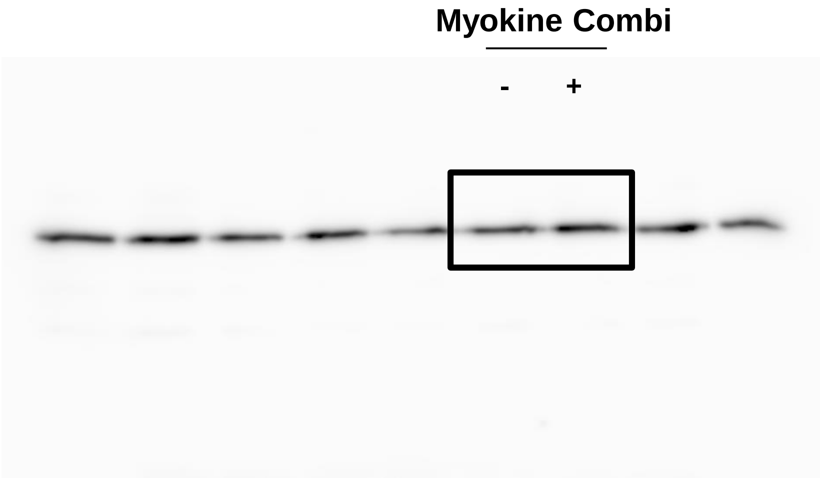

TIFF
cropped part in Figure 5B
flipped horizontally
anti-Casp7 (RRID:AB_2687912)

Figure 6B Panc1

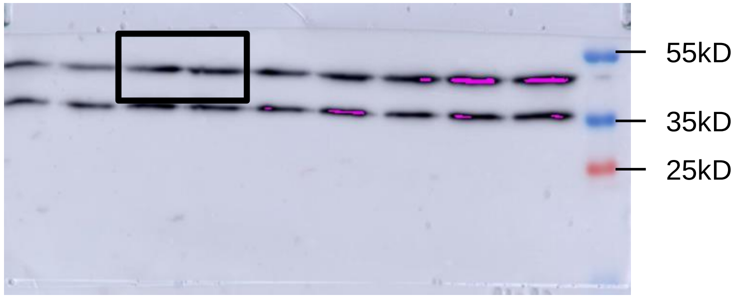

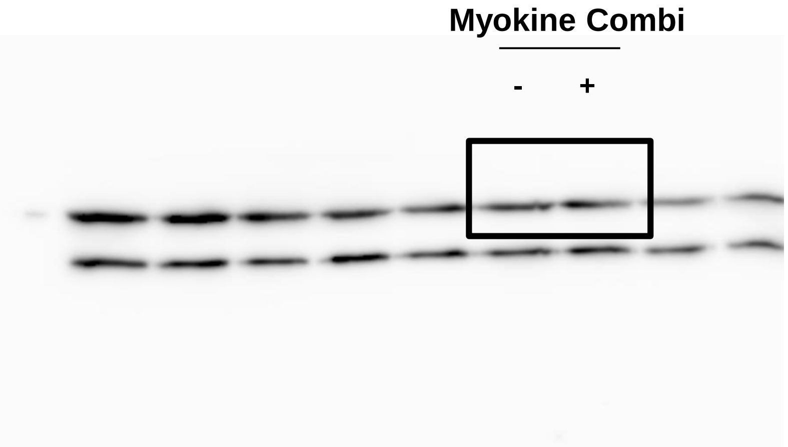

JPEG
anti-Actin (RRID:AB_626632; after anti-Casp7)
with protein marker

TIFF
cropped part in Figure 5B
flipped horizontally
anti-Actin (RRID:AB_626632; after anti-Casp7)

Figure 6B PaCaDD119

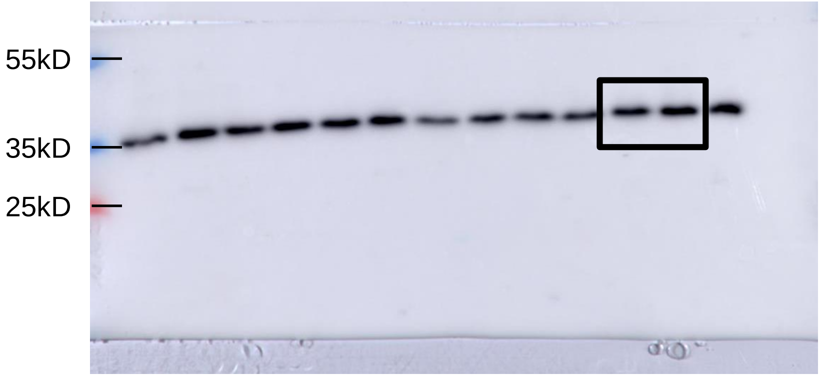

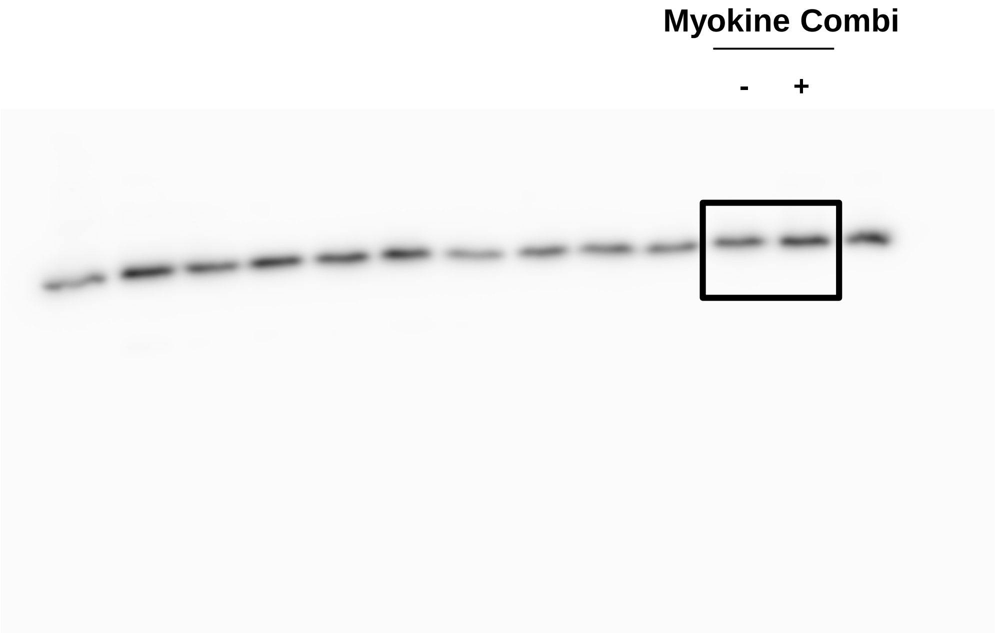

JPEG
anti-Casp7 (RRID:AB_2687912)
with protein marker

TIFF
cropped part in Figure 5B
anti-Casp7 (RRID:AB_2687912)

Figure 6B PaCaDD119

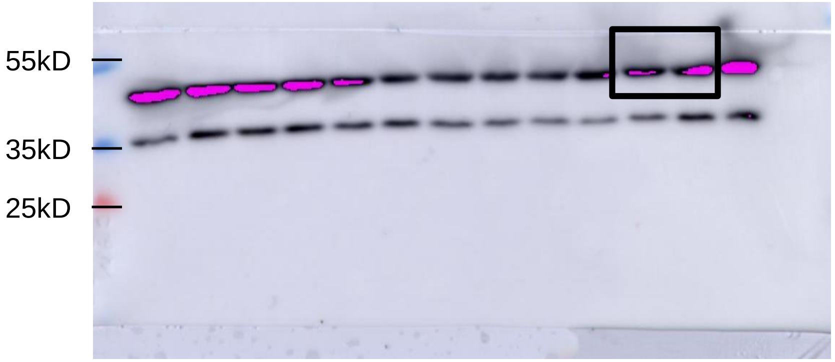

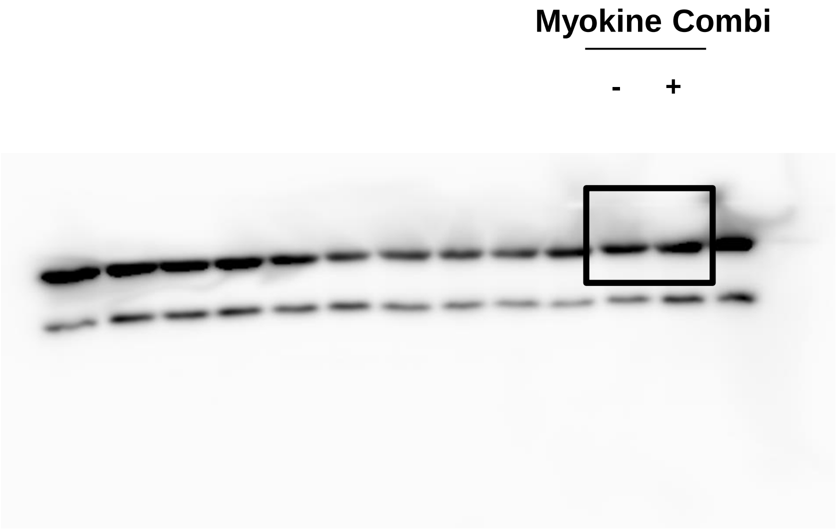

JPEG
anti-Actin (RRID:AB_626632; after anti-Casp7)
with protein marker

TIFF
cropped part in Figure 5B
anti-Actin (RRID:AB_626632; after anti-Casp7)

Figure 6C Panc1

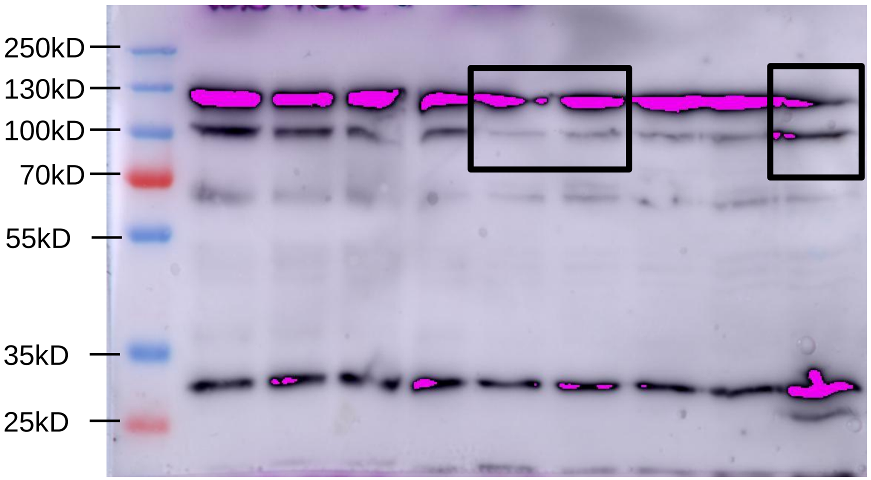

JPEG
anti-PARP (RRID:AB_2160739)
with protein marker

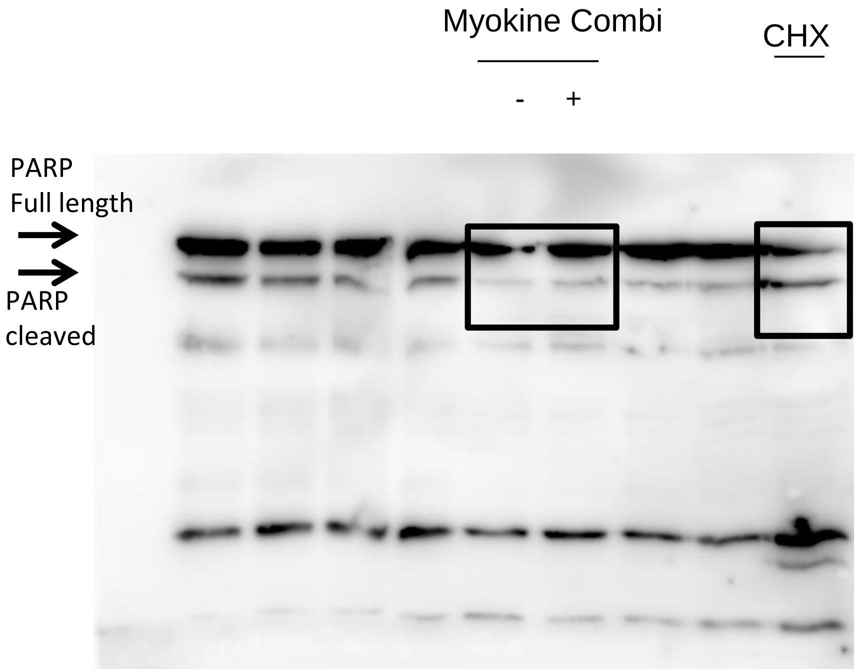

TIFF
cropped parts in Figure 5C
anti-PARP (RRID:AB_2160739)

Figure 6C PaCaDD119

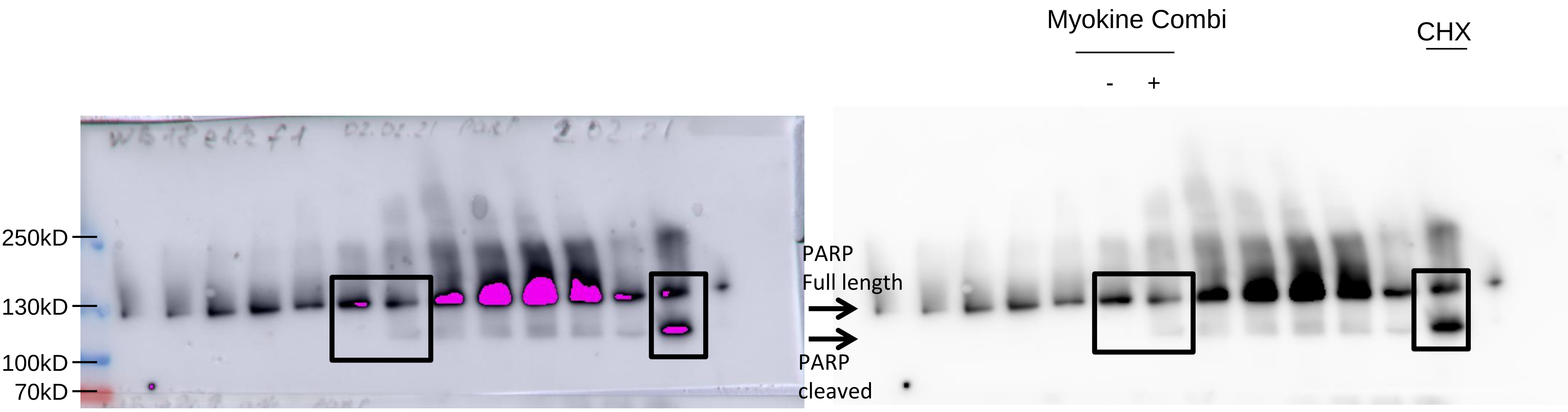

JPEG
anti-PARP (RRID:AB_2160739)
with protein marker

TIFF
cropped parts in Figure 5C
anti-PARP (RRID:AB_2160739)
