# Supplementary material for: Muscle-Derived Cytokines Reduce Growth, Viability and Migratory Activity of Pancreatic Cancer Cells
Source: Cancers (Basel). 2021 Jul 29;13(15):3820. doi: 10.3390/cancers13153820 (PMC8345221; doi:10.3390/cancers13153820)
Supplement: Supplementary file 1 [file cancers-13-03820-s001.zip › cancers-1278687-suppl tables.pdf]

Supplemental Table S1

	Gastric Cancer		
	**Control (n=6)**	**Resistance training using WB-EMS (n=6)**	**P**
**Sex**			-
Male, n (%)	3 (50%)	3 (50%)	
Female, n (%)	3 (50%)	3 (50%)	
**Age** (y)	52.5 ± 13.8	66.3 ± 11.3	0.086 [a]
**Tumor Stage** (UICC)			-
III, n (%)	-	-	
IV, n (%)	6 (100%)	6 (100%)	
**Oncological Therapy**			-
Chemotherapy, n (%)	6 (100%)	6 (100%)	
Other therapies, n (%)	1 (20%)	1 (20%)	
**Karnofsky Index** (%)	80.0 ± 6.3	80.0 ± 12.7	0.862 [b]
**6min-Walking Distance** (m)	595.3 ± 36.9	557.5 ± 71.0	0.426 [b]
**Body Parameters**			
Body Weight (kg)	66.5 ± 16.1	69.5 ± 13.1	0.727 [a]
Weight Loss in the last 3-6 month (%)	7.4 ± 6.5	6.5 ± 4.7	0.667 [b]
Body Mass Index (kg/m^2)	23.7 ± 4.8	24.5 ± 2.0	0.711[a]
Skeletal Muscle Mass (kg)	22.0 ± 6.9	21.4 ± 7.4	0.886 [a]
**Blood Parameters**			
Albumin (g/l)	41.9 ± 3.2	38.8 ± 0.9	0.042 [a]
C-reactive Protein (mg/l)	6.6 ± 9.7	3.5 ± 5.0 (n=5)	0.548 [a]
Creatinine (mg/dl)	0.9 ± 0.3	0.8 ± 0.2	0.466 [a]
Hematocrit (%)	34.4 ± 3.2	35.4 ± 3.5	0.597 [a]
Hemoglobin (g/dl)	11.4 ± 1.5	11.8 ± 1.2	0.646 [a]
Leucocytes (x10^3/µl)	7.5 ± 4.8	4.5 ± 0.6	0.009 [b]
Erythrocytes (x10^6/µl)	4.4 ± 0.9	4.0 ± 0.5	0.589 [b]
Thrombocytes (x10^3/µl)	262.2 ± 86.1	179.8 ± 35.4	0.041 [b]

Pre-intervention characteristics of the patient cohort with gastric cancer. Values are given as mean (SD). Where appropriate, statistical analysis was done using independent samples t-test ([a]) or Mann-Whitney test ([b]). Some significant differences in blood parameters were determined: at study entry, WB-EMS gastric cancer patients had significantly lower serum albumin, and a lower leucocyte and thrombocyte count, compared to non-exercising controls.

Supplemental Table S2 Cytokine Array with plasma from non-exercising control and exercising PC patients

	Normalized data				Ratio post vs. pre				Difference to Ctl plasma pre				Ratio Ex post plasma vs. Ctl post plasma			
Cytokine	Ctl plasma pre	Ctl plasma post	Ex plasma pre	Ex plasma post	Ctl plasma pre	Ctl plasma post	Ex plasma pre	Ex plasma post	Ctl plasma pre	Ctl plasma post	Ex plasma pre	Ex plasma post	Ctl plasma pre	Ctl plasma post	Ex plasma pre	Ex plasma post
Positive																
Positive																
Positive																
Positive																
Negative																
Negative																
ENA78	4441,25	6980,273475	5412,620233	6215,041954	1	1,572	1	1,148	1		1,219	1,399		1		0,890
GCSF	3172,75	2065,361294	2798,226311	3199,270382	1	0,651	1	1,143	1		0,882	1,008		1		1,549
GM-CSF	4441,75	3107,756518	5339,076996	5122,245771	1	0,700	1	0,959	1		1,202	1,153		1		1,648
GRO	13682,25	13219,15275	13205,09261	15989,93928	1	0,966	1	1,211	1		0,965	1,169		1		1,210
GRO-a	6278,25	6574,280277	5557,861647	10475,46895	1	1,047	1	1,885	1		0,885	1,669		1		1,593
I-309	4289,25	2157,263024	6245,605697	1972,089149	1	0,503	1	0,316	1		1,456	0,460		1		0,914
IL-1a	5440,25	3082,473472	8063,306667	3557,526703	1	0,567	1	0,441	1		1,482	0,654		1		1,154
Il-1b	4738,75	3358,234982	7484,379558	4542,930939	1	0,709	1	0,607	1		1,579	0,959		1		1,353
IL-2	2378,75	2089,537009	6326,07587	2821,266796	1	0,878	1	0,446	1		2,659	1,186		1		1,350
IL-3	2495,25	3101,779045	5822,58413	4187,718191	1	1,243	1	0,719	1		2,333	1,678		1		1,350
IL-4	2151,75	2323,500247	3897,5732	3280,921821	1	1,080	1	0,842	1		1,811	1,525		1		1,412
IL-5	3780,25	2722,553041	3194,420827	3765,328729	1	0,720	1	1,179	1		0,845	0,996		1		1,383
IL-6	5629,75	2905,635041	2729,067516	4045,951906	1	0,516	1	1,483	1		0,485	0,719		1		1,392
IL-7	5319,25	3874,975522	5807,777411	6296,512804	1	0,728	1	1,084	1		1,092	1,184		1		1,625
IL-8	5961,75	6753,033041	9295,286697	9687,915179	1	1,133	1	1,042	1		1,559	1,625		1		1,435
IL-10	7538,25	5211,535747	6363,706379	9699,656062	1	0,691	1	1,524	1		0,844	1,287		1		1,861
IL-12	5104,75	1686,483707	6938,303599	2810,754221	1	0,330	1	0,405	1		1,359	0,551		1		1,667
IL-13	5051,75	1587,56145	8015,826832	3106,412373	1	0,314	1	0,388	1		1,587	0,615		1		1,957
IL-15	6365,25	3600,479602	10230,15862	5055,091495	1	0,566	1	0,494	1		1,607	0,794		1		1,404
IFN-gamma	4828,75	4617,274989	10348,25931	5934,642591	1	0,956	1	0,573	1		2,143	1,229		1		1,285
MCP-1	15060,25	19659,11423	23184,96302	26175,49111	1	1,305	1	1,129	1		1,539	1,738		1		1,331
MCP-2	2583,25	3880,986952	4888,045343	4392,030434	1	1,502	1	0,899	1		1,892	1,700		1		1,132
MCP-3	4466,75	3594,796077	4014,24474	4414,821272	1	0,805	1	1,100	1		0,899	0,988		1		1,228
MCSF	7680,75	4237,454983	5023,265192	6540,756486	1	0,552	1	1,302	1		0,654	0,852		1		1,544
MDC	6005,75	5835,399939	6267,669093	8564,758684	1	0,972	1	1,366	1		1,044	1,426		1		1,468
MIG	5531,75	8163,964362	8136,727708	9460,332613	1	1,476	1	1,163	1		1,471	1,710		1		1,159
MIP-1b	10134,75	8558,611157	10199,15085	13220,53639	1	0,844	1	1,296	1		1,006	1,304		1		1,545
MIP-1d	22262,75	22894,81907	30350,84673	22865,87085	1	1,028	1	0,753	1		1,363	1,027		1		0,999
RANTES	61374,25	67713,9146	79900,25734	70906,04785	1	1,103	1	0,887	1		1,302	1,155		1		1,047
SCF	6777,25	4012,695858	8433,53458	6122,108094	1	0,592	1	0,726	1		1,244	0,903		1		1,526
SDF-1	4735,25	4181,0137	8267,992624	4552,996536	1	0,883	1	0,551	1		1,746	0,962		1		1,089
TARC	5465,75	5586,985357	9936,79848	5122,795953	1	1,022	1	0,516	1		1,818	0,937		1		0,917
TGF-b1	4020,25	4721,533176	7474,743145	4988,768893	1	1,174	1	0,667	1		1,859	1,241		1		1,057
TNF-a	4367,25	3515,649916	4079,36808	3862,145053	1	0,805	1	0,947	1		0,934	0,884		1		1,099
TNF-b	10523,75	7796,70579	8638,640852	10589,86278	1	0,741	1	1,226	1		0,821	1,006		1		1,358
EGF	16735,25	23250,43894	13797,1451	13852,78436	1	1,389	1	1,004	1		0,824	0,828		1		0,596
IGF-I	5563,25	7654,855087	7265,504334	10222,88671	1	1,376	1	1,407	1		1,306	1,838		1		1,335
Angiogenin	68189,25	60015,20068	61642,56404	74736,08327	1	0,880	1	1,212	1		0,904	1,096		1		1,245
Oncostatin M	7797,25	4386,190797	8904,983748	4037,798976	1	0,563	1	0,453	1		1,142	0,518		1		0,921
Thrombopoietin	6031,75	3539,402649	9251,761366	4286,917366	1	0,587	1	0,463	1		1,534	0,711		1		1,211
VEGF	8058,75	5677,615118	11358,60899	5820,53605	1	0,705	1	0,512	1		1,409	0,722		1		1,025
PDGF-BB	34224,25	35651,4589	40344,82928	29963,18795	1	1,042	1	0,743	1		1,179	0,875		1		0,840
Leptin	18235,25	20343,69269	18998,10458	14380,61983	1	1,116	1	0,757	1		1,042	0,789		1		0,707
BDNF	30394,75	28149,23741	32761,93891	23994,34738	1	0,926	1	0,732	1		1,078	0,789		1		0,852
BLC	6445,25	7000,837707	7383,226518	6312,923401	1	1,086	1	0,855	1		1,146	0,979		1		0,902
Ck b 8-1	9064,25	5741,667018	6336,226426	6379,429325	1	0,633	1	1,007	1		0,699	0,704		1		1,111
Eotaxin	11546,75	8956,151953	7484,596154	10239,45623	1	0,776	1	1,368	1		0,648	0,887		1		1,143
Eotaxin-2	14960,75	11988,19169	14098,89352	12667,88414	1	0,801	1	0,899	1		0,942	0,847		1		1,057
Eotaxin-3	9496,25	9918,852907	8152,763464	10402,67527	1	1,045	1	1,276	1		0,859	1,095		1		1,049
FGF-4	5597,75	500,7764761	4997,156718	484,0122998	1	0,089	1	0,097	1		0,893	0,086		1		0,967
FGF-6	7522,75	4902,652481	9538,890008	4690,618118	1	0,652	1	0,492	1		1,268	0,624		1		0,957
FGF-7	5091,25	2872,061901	7505,492505	2977,354691	1	0,564	1	0,397	1		1,474	0,585		1		1,037
FGF-9	6077,75	4427,456874	8110,279298	3656,741748	1	0,728	1	0,451	1		1,334	0,602		1		0,826
Flt-3 Ligand	7284,75	5600,110813	8600,053383	5374,828006	1	0,769	1	0,625	1		1,181	0,738		1		0,960
Fractalkine	5523,25	5972,292623	6820,755622	4891,101957	1	1,081	1	0,717	1		1,235	0,886		1		0,819
GCP-2	5550,25	7734,403584	7512,421982	6496,135962	1	1,394	1	0,865	1		1,354	1,170		1		0,840
GDNF	6426,25	7798,265328	7128,049732	6136,42402	1	1,214	1	0,861	1		1,109	0,955		1		0,787
HGF	8603,75	8611,073266	6233,81888	7658,261977	1	1,001	1	1,229	1		0,725	0,890		1		0,889
IGFBP-1	10088,25	11416,30969	9245,43929	10314,08257	1	1,132	1	1,116	1		0,916	1,022		1		0,903
IGFBP-2	19246,75	19626,31509	18595,49741	18160,13046	1	1,020	1	0,977	1		0,966	0,944		1		0,925
IGFBP-3	11143,75	5696,003518	10804,96268	3599,116025	1	0,511	1	0,333	1		0,970	0,323		1		0,632
IGFBP-4	5413,25	2776,012311	6782,646847	2622,141943	1	0,513	1	0,387	1		1,253	0,484		1		0,945
IL-16	5209,25	3387,606412	7317,029818	3309,121679	1	0,650	1	0,452	1		1,405	0,635		1		0,977
IP-10	7463,75	7192,193888	10429,84834	6056,131566	1	0,964	1	0,581	1		1,397	0,811		1		0,842
LIF	16831,75	12270,66312	14906,9788	10969,73811	1	0,729	1	0,736	1		0,886	0,652		1		0,894
LIGHT	4544,75	5601,389162	5630,412617	4588,228823	1	1,232	1	0,815	1		1,239	1,010		1		0,819
MCP-4	4542,75	6438,159815	4593,960825	4552,315677	1	1,417	1	0,991	1		1,011	1,002		1		0,707
MIF	7201,25	9383,073714	7186,239912	7555,074714	1	1,303	1	1,051	1		0,998	1,049		1		0,805
MIP-3a	5987,75	7437,345404	5470,810984	5818,169836	1	1,242	1	1,063	1		0,914	0,972		1		0,782
NAP-2	29937,75	30823,77718	31886,2887	33750,05738	1	1,030	1	1,058	1		1,065	1,127		1		1,095
NT-3	13407,25	11609,04883	9459,748354	9785,348233	1	0,866	1	1,034	1		0,706	0,730		1		0,843
NT-4	8089,25	2117,405154	7707,546109	2038,168233	1	0,262	1	0,264	1		0,953	0,252		1		0,963
Osteopontin	7039,75	4148,221494	6954,517246	3707,1989	1	0,589	1	0,533	1		0,988	0,527		1		0,894
Osteoprogeterin	4832,75	2625,174408	6310,253455	2942,729066	1	0,543	1	0,466	1		1,306	0,609		1		1,121
PARC	4633,25	3602,941221	6643,045097	3174,844101	1	0,778	1	0,478	1		1,434	0,685		1		0,881
PIGF	3952,25	3335,378775	5026,35982	2332,574438	1	0,844	1	0,464	1		1,272	0,590		1		0,699
TGF-b2	29582,25	19715,78683	23078,09262	21582,44575	1	0,666	1	0,935	1		0,780	0,730		1		1,095
TGF-b3	4495,25	6190,313989	3903,031574	3870,631687	1	1,377	1	0,992	1		0,868	0,861		1		0,625
TIMP-1	30448,75	30667,51711	34948,66147	37281,09886	1	1,007	1	1,067	1		1,148	1,224		1		1,216
TIMP-2	23281,25	20307,45529	17715,16139	17606,26655	1	0,872	1	0,994	1		0,761	0,756		1		0,867
Positive																
Positive																

Supplemental Table S3 Cytokine Array with medium from human myotubes after EPS

Cytokine	normalised data		Ratio 2x20min EPS vs. Untreated	
Positive				
Positive				
Negative				
Negative				
Blank				
Angiogenin	83773,5	619150,955	1	**7,390773397**
BDNF	1049635	2710016,99	1	**2,581866064**
BLC	558938	-221358,898		
BMP4	1003927	1260848,22	1	**1,255916233**
BMP6	124959	-158618,45		
CCL23	94698,5	514774,89	1	**5,435934996**
CNTF	-112081	344609,161		
EGF	-137854,5	129063,194		
Eotaxin1	284559,5	269042,584	1	**0,9454704**
Eotaxin2	778702,5	717815,73	1	**0,921809973**
Eotaxin3	492682,5	560116,293	1	**1,136870688**
FGF6	4459851	3574430,88	1	**0,801468677**
FGF7	375328	428847,044	1	**1,142592729**
Flt3 Ligand	123983,5	268316,84	1	**2,164133455**
Fractalkine	105138	184690,015	1	**1,756643789**
GCP2	400358	1623799,1	1	**4,055867748**
GDNF	324442,5	521181,584	1	**1,60639122**
GMCSF	-145982	41166,8574		
I-309	743043,5	642132,646	1	**0,864192535**
IFNgamma	150462	92608,5143	1	**0,615494373**
IGFBP1	117965,5	185684,575	1	**1,574058307**
IGFBP2	3446163,5	2330753,25	1	**0,676332753**
IGFBP4	865435,5	481167,967	1	**0,555983626**
IGF1	206499	177443,497	1	**0,859294705**
IL10	121499	173976,189	1	**1,431914572**
IL13	-473103	165320,66		
IL15	858831	671013,131	1	**0,781309863**
IL16	441859,5	242266,514	1	**0,548288572**
IL1alpha	638477	601831,402	1	**0,942604671**
IL1beta	412407,5	440414,651	1	**1,067911351**
IL1Ralpha	1363152,5	1208601,32	1	**0,88662224**
IL2	24040,5	58344,4168	1	**2,426921938**
IL3	676526,5	401246,927	1	**0,59309861**
IL4	1049808,5	897133,358	1	**0,854568579**
IL5	12410	79462,5963	1	**6,403110096**
IL6	6741780	10011162,4	1	**1,484943503**
IL7	-91173,5	-64633,3891		
Leptin	5311553,5	4769969,11	1	**0,898036538**
LIGHT	655455	1531362,01	1	**2,336334316**
MCP1	28329811,5	31822742,9	1	**1,123295258**
MCP2	382371,5	277988,775	1	**0,727012277**
MCP3	566078	702192,816	1	**1,240452405**
MCP4	884939,5	814215,286	1	**0,920080171**

**MCSF**	1246736,5	1119506,54	**1**	**0,897949597**
**MDC**	1076801	924770,491	**1**	**0,858812808**
**MIG**	1144	121036,926	**1**	**105,8015089**
**Mip1delta**	508232	429967,214	**1**	**0,846005788**
**MIP3alpha**	242945	315291,167	**1**	**1,297788251**
**NAP2**	538777	391139,91	**1**	**0,725977371**
**NT3**	5353980,5	4896562,29	**1**	**0,914564835**
**PARC**	492281	685912,121	**1**	**1,393334541**
**PDGFBB**	3757851,5	3182645,63	**1**	**0,846932251**
**RANTES**	1450387,5	1495571,79	**1**	**1,031153251**
**SCF**	3037663	2516464,82	**1**	**0,828421331**
**SDF1alpha**	691202,5	818515,744	**1**	**1,184190949**
**TARC**	2275165	2007252,25	**1**	**0,882244695**
**TGFbeta1**	574393,5	531834,729	**1**	**0,925906593**
**TGFbeta3**	263082,5	271477,103	**1**	**1,031908635**
**TNFalpha**	-23104	76235,5841		
**TNFbeta**	2021083,5	1690299,94	**1**	**0,836333553**
**blank**	-235666	-131421,255		
**blank**	-343672	-21326,6185		
**blank**	-336031	54594,9418		
**blank**	-298381	-54441,419		
**Positive**	45766740,5	57862378,1		
**Positive**				
**Positive**				
**Negative**				
**Negative**				
**blank**				
**Acrp30**	512614	314971,612	**1**	**0,614442079**
**AgRP**	784364	685497,983	**1**	**0,873953907**
**ANGPT2**	1410420	1230785,12	**1**	**0,872637315**
**AREG**	836825,5	750792,269	**1**	**0,897190955**
**Axl**	-225149,5	326301,795		
**bFGF**	1097222,5	1249769,68	**1**	**1,139030305**
**bNGF**	245431	304670,309	**1**	**1,241368485**
**BTC**	369725,5	496433,801	**1**	**1,34270912**
**CCL28**	275019,5	266934,747	**1**	**0,970602981**
**CTACK**	3436742	3993422,91	**1**	**1,161979255**
**Dtk**	779955	957860,184	**1**	**1,228096728**
**EGFR**	409518	498926,951	**1**	**1,218327279**
**ENA78**	1369057,5	959609,425	**1**	**0,700927043**
**Fas**	1807167,5	1513190,08	**1**	**0,837326968**
**FGF4**	194524,5	-86905,5898		
**FGF9**	2716279,5	2843093,29	**1**	**1,046686576**
**GCSF**	-138436	11191,3017		
**GITR ligand**	727934,5	900747,446	**1**	**1,23740178**
**GITR**	825715	1002341,86	**1**	**1,213907776**
**Gro**	9925303,5	25008798,3	**1**	**2,51970112**
**Groalpha**	1961736	6594589,84	**1**	**3,361609227**
**HCC4**	1132426	1074528,83	**1**	**0,948873331**

**HGF**	330650	437145,564	**1**	**1,322079432**
**ICAM1**	834492	762957,187	**1**	**0,914277414**
**ICAM3**	258311	421875,262	**1**	**1,633206723**
**IGFBP3**	821593	931289,012	**1**	**1,133516244**
**IGFBP6**	824294	1106021,43	**1**	**1,341780271**
**IGF1 sR**	567853,5	509932,275	**1**	**0,897999705**
**IL1 R4**	451872,5	745392,687	**1**	**1,649564174**
**IL1 R1**	799938	887373,917	**1**	**1,109303368**
**IL11**	449695,5	593048,162	**1**	**1,318777177**
**IL12 p40**	2047835	2265671,04	**1**	**1,106373826**
**IL12 p70**	857684	1013444,26	**1**	**1,181605647**
**IL17**	647133	767694,076	**1**	**1,186300306**
**IL2 Ralpha**	891631	790027,662	**1**	**0,886047773**
**IL6 R**	457555,5	569726,543	**1**	**1,245152867**
**IL8**	24765979	35226616,8	**1**	**1,422379337**
**ITAC**	495882	1031534,01	**1**	**2,080200553**
**XCL1**	673951	690341,556	**1**	**1,0243201**
**MIF**	1461133	2021288,54	**1**	**1,383370671**
**Mip1alpha**	1196318	1407361,45	**1**	**1,176410828**
**Mip1beta**	1863461,5	2220629,52	**1**	**1,191669114**
**Mip3beta**	2882942	3136711,66	**1**	**1,088024545**
**MSPalpha**	390058	459178,319	**1**	**1,177205234**
**NT4**	375027	460019,781	**1**	**1,226631099**
**OPG**	5254914,5	6524580,29	**1**	**1,241614928**
**OSM**	3831861	3569026,62	**1**	**0,931408166**
**PLGF**	786736	882225,188	**1**	**1,121373864**
**sgp130**	1183795	1314040,37	**1**	**1,110023586**
**sTNFRII**	409574	503307,263	**1**	**1,228855501**
**sTNFRI**	933734,5	1178150,73	**1**	**1,261762022**
**TECK**	586985,5	671883,213	**1**	**1,144633407**
**TIMP1**	5501148	6690267,11	**1**	**1,216158356**
**TIMP2**	2143805	3145883,64	**1**	**1,46742994**
**THPO**	467462,5	590756,367	**1**	**1,263751354**
**TRAIL R3**	29807,5	341920,504	**1**	**11,47095544**
**TRAIL R4**	199480,5	315339,721	**1**	**1,580804747**
**uPAR**	186476,5	257506,623	**1**	**1,380906563**
**VEGF**	436536,5	711199,82	**1**	**1,629187525**
**VEGF D**	-195940,5	-101699,176		
**blank**	-443995	-51297,4706		
**blank**	-280091	11301,3501		
**blank**	-338365	197288,02		
**blank**	-350996	263733,25		
**Positive**	16652214	25784989,2		
